# New Data Set of Polychlorinated Dibenzo-*p*-dioxin and Dibenzofuran Half-Lives: Natural Attenuation and Rhizoremediation Using Several Common Plant Species in a Weathered Contaminated Soil

**DOI:** 10.1021/acs.est.0c01857

**Published:** 2020-07-20

**Authors:** Elisa Terzaghi, Lorenzo Vergani, Francesca Mapelli, Sara Borin, Giuseppe Raspa, Elisabetta Zanardini, Cristiana Morosini, Simone Anelli, Paolo Nastasio, Vanna Maria Sale, Stefano Armiraglio, Antonio Di Guardo

**Affiliations:** † DiSAT, University of Insubria, Via Valleggio 11, Como 22100, Italy; ‡ DeFENS, University of Milan, Via Celoria 2, Milan 20133, Italy; § DICMA, Sapienza University of Rome, Via Eudossiana 18, Rome 00184, Italy; ∥ ERSAF, Via Pola 12, Milan 20124, Italy; ⊥ Municipality of Brescia—Museum of Natural Sciences, Via Ozanam 4, Brescia 25128, Italy

## Abstract

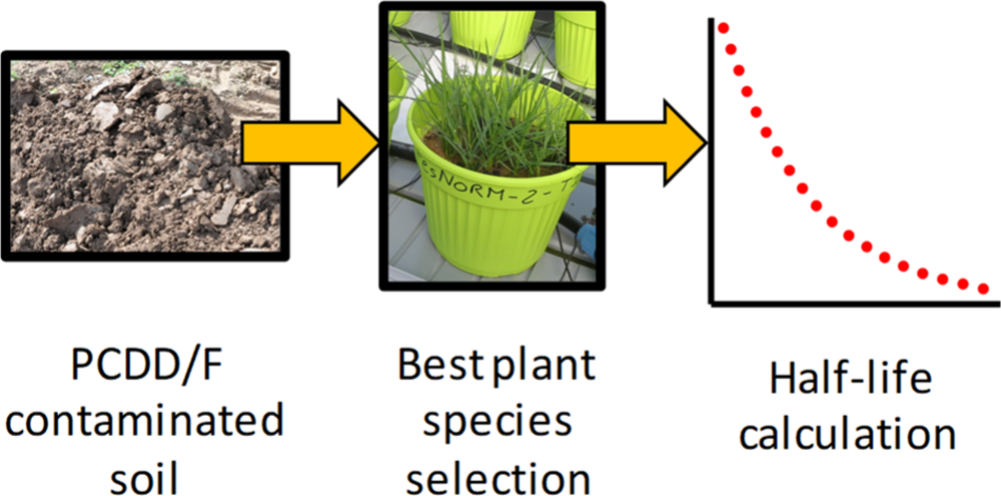

In this paper, a new data set of polychlorinated dibenzo-*p*-dioxin and dibenzofuran (PCDD/Fs) half-lives (HLs) in soil is presented. Data are derived from a greenhouse experiment performed with an aged contaminated soil under semi-field conditions, obtained from a National Relevance Site (SIN) located in Northern Italy (SIN Brescia-Caffaro). Ten different treatments (combination of seven plant species with different soil conditions) were considered together with the respective controls (soil without plants). The ability of the plants to stimulate the biodegradation of these compounds was evaluated by measuring the PCDD/F concentration reduction in soil over a period of 18 months. The formation of new bound residues was excluded by using roots as a passive sampler of bioaccessible concentrations. The best treatment which significantly reduced PCDD/F concentrations in soil was the one with *Festuca arundinacea* (about 11–24% reduction, depending on the congener). These decreases reflected in HLs ranging from 2.5 to 5.8 years. Simulations performed with a dynamic air-vegetation-soil model (SoilPlusVeg) confirmed that these HLs were substantially due to biodegradation rather than other loss processes. Because no coherent PCDD/F degradation HL data sets are currently available for soil, they could substantially improve the predictions of soil remediation time, long-range transport, and food chain transfer of these chemicals using multimedia fate models.

## 1 Introduction

Polychlorinated dibenzo-*p*-dioxins (PCDDs) and furans (PCDFs) are two classes of chlorinated organic compounds, generally classified as undesired and toxic byproducts released by different human activities, industrial processes, and natural sources.^1,2^ They include 210 possible congeners, among which, 7 PCDD and 10 PCDF congeners were identified as toxic for humans and wildlife.^3,4^ PCDD/F concentrations in soil range from sub pg I-toxic equivalency (TEQ) g^–1^ dw level in remote sites,^5^ to few tens of pg I-TEQ g^–1^ dw in background samples^6^ and up to a few hundreds of pg I-TEQ g^–1^ in urban/industrial soils.^7^ However, higher concentrations were found in incident sites such as that of Seveso in Italy^8^ and superfund sites in the US.^9^

Contaminated soils represent a secondary source of PCDD/Fs and therefore their remediation is fundamental to reduce their concentrations and to stop their continuous input in the ecosystems and food chains.^10^ Nowadays, several physical, thermal, chemical, and biological strategies are available to remediate PCDD/F contaminated soils, including bioremediation technologies.^11−14^ Among bioremediation techniques, rhizoremediation (RR) is a type of phytoremediation that exploits the plant biostimulation effect on the degradative microbial populations which naturally evolved in polluted soils.^15^ However, although many RR studies were performed for polychlorinated biphenyls (PCBs),^15,16^ very few attempts are known for the remediation of PCDD/F-contaminated soils.^17−21^

One of the most important PCDD/F contaminated site in Europe is the national priority site for remediation (SIN) Brescia-Caffaro (Italy), in which surface water, contaminated by the Caffaro factory (former producer of PCBs and other chemicals), was used to irrigate adjacent agricultural areas for about 50 years.^22^ This resulted in about 100 ha of agricultural areas being contaminated by PCBs, PCDDs, PCDFs, dichlorodiphenyltrichloroethane (DDT), metals, and metalloids, such as Hg and As, at concentrations often exceeding the legal thresholds.^22,23^ More specifically, PCDD/F concentrations reached values between 19 and 60 pg WHO05-TEQ g^–1^ (e.g., 26–77 pg I-TEQ g^–1^)^22^ and the highest contribution to TEQ was mostly due to PCDFs, well-known impurities of PCB production.^24^

In this study, we set up a RR experiment of ∼18 months in a greenhouse, aiming to identify the best plant species (including also some of the most common grass species) and soil conditions which induced an efficient microbial degradation of PCDD/F in soil. In order to reproduce realistic semi-field conditions, the experiment was conducted in large pots containing weathered PCDD/F-contaminated soil from the SIN Brescia-Caffaro site following a robust experimental design in terms of replicates and controls, as described in a previous work of our group,^25^ to overcome some of the weaknesses and limitations of RR experiments performed so far.^16^ The results allowed to obtain the first (to the best of our knowledge) complete and coherent experimental data set of degradation half-lives (HLs) of PCDD/Fs in soil, obtained combining natural attenuation (NA) and RR. The data set could be later utilized to predict field remediation times using available fate models and ecological scenarios^26−32^ to derive, for example, better estimates of long range transport as well as food chain contamination.^33−35^

## 2 Materials and Methods

### 2.1 Experimental Design

Roughly 2500 kg of soil were collected from the SIN Brescia-Caffaro, located in Northern Italy^22^ and homogenized as described in previous paper.^25^ Seven plant species were used in the present greenhouse study, that is, *Phalaris arundinacea* L. (reed canarygrass), *Festuca arundinacea* Schreb. (tall fescue), *Cucurbita pepo* L. ssp pepo (pumpkin), *Medicago sativa* L. (alfalfa), *Brassica juncea* (L.) Czern. (brown mustard), *Salix caprea* L. (goat willow), and *Athyrium filix-foemina* (L.) Roth (lady fern). These plants were among the most commonly employed species in bioremediation studies and some among the most common grass species. They were cultivated under five different cropping conditions, namely, “only plant”, “redox cycle”, “consociation”, “compost addition”, and “ammonium thiosulfate addition” to investigate the effect of 10 different treatments in the RR of PCDD/Fs. Seven controls (i.e., contaminated soil not planted but with the same soil conditions as the treatments and not contaminated planted soil) were also set up. For a full description of the 10 treatments (P) and 7 controls (C) please refer to a previous paper^25^ and Table 1. Briefly, the different controls were designed to reflect specific conditions of the related treatment(s); for example, the treatment P6 could not be compared to the general control C2 because it required the addition of compost to evaluate the specific variance induced by this additive factor. In order to reduce the sampling variability, we implemented a number of measures such as the Japanese slab cake technique,^36^ an incremental sampling procedure, employed each time a sample volume reduction was needed, from soil collection in the field to soil preparation for potting, sample preparation for storage, and at sample extraction time. Individual polypropylene pots (top diameter: 260 mm; height: 215 mm; soil holding capacity: 8.2 l) filled with approximately 6 kg of soil were used. The experiment lasted for 18 months (from May 13, 2015 to Nov 16, 2016). Five sampling times were considered: 0 (T0), 3 (T1), 6 (T2), 12 (T3), and 18 (T4) months. At each sampling time, soil and plant biomass (roots and shoots) were sampled for microbiological and chemical analyses as described previously.^25^ Only T0, T2, and T4 samples were analyzed for chemical analyses. The experiment was performed in triplicate, that is, three pots for each treatment and control at each time were sacrificed for analysis. For more details about greenhouse and pot conditions (e.g., illumination, temperature, irrigation, and fertilization), refer to ref (25). Briefly, the greenhouse used in the experiment was not heated during the winter season to reflect the seasonal variability while it was partially open during summer to avoid excessive temperature. This was done to simulate more realistic semi-field conditions and reflect the air temperature seasonal variability in Brescia. The moisture level was maintained at about 30% of the soil total volume in all pots by drip irrigation.

**Table 1:** Treatments (P) and Relative Controls (C) in the Greenhouse Experiment

plant species	treatments	controls
*P. arundinacea*	P1—only plant	C2—no plant with fertilizer
	P2—redox cycle	C3—no plant with fertilizer and redox cycle
*F. arundinacea*	P3—only plant	C2—no plant with fertilizer
	P4—consociation with *C. pepo* ssp. pepo	C2—no plant with fertilizer
	P5—redox cycle	C3—no plant with fertilizer and redox cycle
	P6—compost addition	C5—no plant with fertilizer and compost
*M. sativa*	P7—only plant	C2—no plant with fertilizer
*S. caprea*	P8—consociation with *A. filix-foemina*	C7—no plant with fertilizer and additional soil^a^
*B. juncea*	P9—only plant	C2—no plant with fertilizer
	P10—ammonium thiosulfate addition	C4—no plant with fertilizer and thiosulfate
		C1—no plant no fertilizer
		C6—uncontaminated soil with plant and fertilizer

a In this control, given *A. filix-foemina* seedling transplant, additional soil was included to compensate for soil added in the corresponding treatment.

### 2.2 PCDD/F and Organic Carbon Determination in Soil Samples

PCDD/Fs were analyzed, according to an EPA 1613 B (1994)^37^ method, by a commercial laboratory (INDAM, Castel Mella BS, Italy). In brief, samples were extracted with an accelerated solvent extractor (ASE) (Thermo Scientific Dionex ASE 350) and analyzed with HRGC/HRMS (Thermo Scientific TRACE GC Ultra coupled with Thermo Scientific DFS MS). Seventeen congeners and PCDD/F classes were determined. The 17 congeners measured were those with Toxicity Equivalency Factor (TEF).^38^ For more details on the selected congeners and analytical methods, please see Supporting Information S1. Concentrations of PCDD/Fs will be reported as individual congener concentrations, which means not translated in TEQ using TEF values, unless reported. Organic carbon (OC) in soil samples was determined, according to the UNI EN 13137 method (2002)^39^ (after the removal of carbonates), with an elementar analyzer Elementar vario MACRO cube [Elementar Analysensysteme GmbH, Langenselbold (Germany)].

### 2.3 Quality Assurance/Quality Control

Limits of quantitation (LOQs) ranged between 0.1 and 0.5 pg g^–1^ dw, depending on congeners. A certified referenced material (BCR529 Industrial Soil, EC JRC, Ispra, Italy) was used to monitor method performance. Laboratory blanks (Thermo ASE Prep DE Diatomaceous Earth) were included at the ratio of 3 every 20 samples. Sampling blanks (background contaminated soil collected in the village of Tignale, about 100 km away from Brescia) were used at each sampling time to assess cross-contamination during the sampling procedure. A specific control (C6) was set up to monitor the soil volatilization-driven cross-contamination in the greenhouse. Single congener PCDD/F concentrations in laboratory and sampling blanks, as well as in C6 samples were generally lower than LOQ; additionally, also the sum of the 17 TEF PCDD/F concentrations in blanks and C6 samples was never higher than 1% of the concentration measured in the soil samples indicating that cross-contamination via air as well as sampling/analytical phases did not significantly alter the concentrations in the samples. The metrological service of the Italian National Institute for Environmental Protection and Research (ISPRA) was involved in the validation of the entire procedure for the PCDD/F analysis, including precision, repeatability, and uncertainty of the measurements.^40^

### 2.4 Calculation of Concentration Reduction in Time

Three different conditions were considered:^25^ (1) NA and plant-independent stimulation (NA), that is, the effect of volatilization, infiltration, and biodegradation stimulated by fertilization and irrigation, (2) RR, that is, the effect of enhanced biodegradation because of plant–microbe interactions, and (3) the combined effect of NA plus RR (OVERALL, NA + RR). PCDD/F concentration reduction because of NA, RR, and NA plus RR (NA + RR) was calculated comparing concentration of relative controls (*C*) and treatments (*P*) at different times (T0, T2, T4) as follows
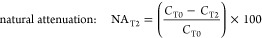
1or
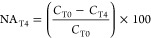
2
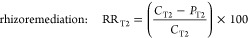
3or
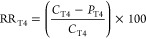
4
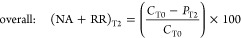
5or
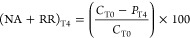
6

These calculations were performed for each of the 17 PCDD/F congeners for all treatments and the PCDD/F classes for P3 samples.

### 2.5 Calculation of the Degradation Rate for RR

Degradation rates (*k*_D_) and HLs were calculated for the 17 PCDD/F congeners (and PCDD/F classes for P3 samples) assuming a first order kinetic disappearance, considering soil concentrations in control pots at T0 (*C*_T0_) and in treatment pots at T4 (*P*_T4_) and the duration of the experiment (553 days), as follows
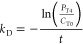
7and
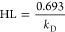
8

HLs were temperature-adjusted to a reference temperature of 25 °C using the Q10 rule^41^ because the average temperature in the greenhouse was 19 °C.

### 2.6 Soil Pore-water Concentration Calculation

In order to assess whether PCDD/F bound residues were to be accounted in the measured levels, the concentrations of all PCDD/Fs in soil pore-water were estimated in two different ways:

(1) (*C*_pw(1)_) were calculated from concentrations in soil and soil-water partition coefficients

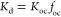
9
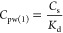
10where *K*_d_ (L kg^–1^) is the soil–water partition coefficient, *K*_oc_ (L kg^–1^) is the OC–water partition coefficient^42^ (*K*_oc_ = 0.41*K*_ow_), *f*_oc_ is the OC fraction in soil (0.018), *C*_s_ is the concentration in soil at T4 of treatment P3 (tall fescue) (ng kg^–1^ dw), and *C*_pw(1)_ is the estimated soil pore-water concentration (ng L^–1^) obtained accordingly.

(2) (*C*_pw(2)_) were obtained dividing the concentrations measured in roots with the root concentration factors

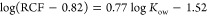
11
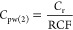
12where RCF is the root concentration factor (L kg^–1^ ww) estimated according to an existing equation for barley^43^ and *C*_r_ is the concentration in roots on a wet weight basis at T4 of treatment P3 (tall fescue) (ng kg^–1^ ww), considering a water fraction of 0.80, and *C*_pw(2)_ is the estimated soil pore-water concentration (ng L^–1^) obtained accordingly.

### 2.7 SoilPlusVeg Model Simulations

A number of one-year simulations were performed with the SoilPlusVeg model^29,30^ in order to investigate the influence of soil temperature and water content on PCDD/F degradation rates. 2,3,7,8-TCDD was chosen as the reference chemical. Two different scenarios (SIM1 and SIM2) were used: in SIM1 the seasonal variability of temperature and rainfall of a city like Brescia was considered and SIM2 was as the previous scenario but without rainfall. Additionally, the model was used to estimate the contribution of degradation, volatilization, infiltration, and root uptake to PCDD/F disappearance from soil for 3 PCDD/F congeners of different physico-chemical properties, that is, 2,3,7,8-TCDD, OCDD, 1,2,3,4,7,8-HxCDF, to evaluate the importance of other loss processes. For more details about simulation scenarios see Supporting Information S1.

### 2.8 Soil Microbiological Analysis

The structure of the bacterial and fungal communities inhabiting the soil samples collected at the end of the RR experiment was studied applying the automated ribosomal intergenic spacer analysis (ARISA) DNA fingerprinting method.^25^ Each polymorphic ARISA peak was defined as a different operational taxonomic unit (OTU). Ecological α-diversity indices were calculated from the ARISA data set (quantitative matrix of the OTUs within each sample) using PAST software.^44^ The considered α-diversity indices are (i) richness, expressed as the number of OTUs, (ii) diversity, expressed by the Shannon index, and (iii) evenness. Soil samples were also previously analyzed to assess the soil hydrolytic activity (a proxy of OC degradation), the β-diversity in terms of bacterial and fungal communities and the abundance of bacteria and fungi.^25^ These data are discussed here in comparison to the observed PCDD/F degradation.

### 2.9 Statistical Analysis

All statistical analyses regarding PCDD/F concentration reductions were performed with XLSTAT software by Addinsoft SARL (version 2019.2.3, Boston, USA). The data were subjected to the Student’s *t*-test (α = 0.05) when its validity conditions (normal distribution and equal variance) were satisfied. When the equal variance test failed, the Welch’s *t*-test (α = 0.05) was performed, while when both normality and equal variance test failed the nonparametric Mann–Whitney test was used. Significant differences in ecological indices between each treatment and the corresponding control were obtained with the Student’s *t*-test (α = 0.05) after confirming its validity conditions using the R software version 3.6.3.^45^

## 3 Results and Discussion

### 3.1 Environmental Parameters and Starting Conditions

A temporal trend of the monitored environmental parameters, such as temperature in the greenhouse, soil water content, redox potential, and OC content in control and treatment pots, is fully discussed elsewhere.^25^ The OC content in the soil ranged from 1.6 to 1.8% in all control and treatment pots apart from C5 and P6 (4.3–5.7%) due to compost addition and of C7 and P8 (1.6–2%) due to the addition of the soil necessary to transplant *A. filix-foemina* plants. The total PCDD/F concentration (for the 17 congeners) at the beginning of the experiment (T0) in the control soil (unplanted and unfertilized, C1 to C4) was 5472 pg g^–1^ dw on average corresponding to 252 pg WHO05-TEQ g^–1^ dw (Figure S1). PCDFs prevailed on PCDDs and the most abundant congeners were 2,3,7,8-TCDF, 2,3,4,7,8-PCDF, and 1,2,3,4,7,8-HxCDF (around 50–70 pg WHO05-TEQ g^–1^ dw). While PCDDs with TEF represented about 80% of total PCDDs, PCDFs with TEF representing about 50% of the total PCDFs (Figure S2). C5 and C7 showed lower PCDD/F concentrations (5135 and 5233 pg g^–1^ dw, respectively) because of the dilution given by the addition of compost and other soil amendments (Table S4). This was also confirmed by the different OC content: 17 g kg^–1^ dw in C1–C4, 52 g kg^–1^ dw in C5, and 20 g kg^–1^ dw in C7. Although the total PCDD/F concentration did not significantly differ among controls, some congeners measured in C5 did significantly differ from C1–C4 (0.02 < *p* < 0.05) and C7 (0.0003 < *p* < 0.05) (Table S5). For this reason, only the concentrations of P6 and its control C5 were normalized to OC. Concentrations in the uncontaminated control (C6) were more than two orders of magnitude lower (35 pg g^–1^ dw) and OC was 23 g kg^–1^ dw (Table S4).

### 3.2 Impact of the Treatments on the α-Diversity of the Soil Microbial Communities

We previously studied the β-diversity of the soil microbial communities associated with the different treatments and their correspondent controls by means of DNA-based fingerprinting, reporting that the use of the selected plants and conditions solely and significantly impacted the bacterial community structure, while the fungal one could not be discriminated according to the applied treatment at T4.^25^ The data set obtained applying the DNA-based fingerprinting was further analyzed to describe the α-diversity of the microbial communities within each treatment and control. The values of the considered α-diversity indices (i.e., richness, diversity, and evenness) are shown in Figure S3, while Table S6 indicates the results of the statistical comparison between the index values in each treatment and the related control. Differently from what was previously observed studying the β-diversity, α-diversity indices revealed an effect of some of the treatments also on the soil fungal communities. In particular, the diversity (i.e., Shannon index) of the soil fungal community in the treatments P1, P3, P4, and P7 was significantly higher than that of the fungal community in the related unplanted control soil (C2). These data suggest that the plants and growth conditions applied in these treatments exert an influence on the soil fungal community and shape its composition, promoting a higher phylogenetic diversity. Accordingly, also Evenness values of the fungal communities were higher in these treatments compared to the C2 control, although such a difference was statistically significant only for the P1 treatment. Considering the soil bacterial communities, all the plant and soil treatment combinations showed at least one index significantly different from the unplanted C2 control, suggesting a strong plant influence in particular on the bacterial fraction of the soil microbiota. The diversity values were significantly different in the treatments P1, P8, and P9. Previous studies on RR of polluted soils showed a positive effect of the plant on the soil bacteria diversity, which increased with the depletion of the contaminants.^46−48^ However, these studies were performed using the soil recently affected by contamination events^46,48^ or sterilized soil,^47^ while we considered a historically polluted site where the autochthonous microbiota is well adapted and selected by the high levels of multiple, persistent contaminants.^22,49^ Possibly, the relatively short duration of our experiment may have not been enough to show a significant detoxification effect and a subsequent increase of the bacterial diversity. Instead, we unveiled a general effect of the planted treatments on the α-diversity of the soil bacterial communities analyzing the richness values. In fact, all the treatments apart from P8 and P9 showed a significantly lower richness value compared to their correspondent unplanted control. This result, in agreement with previous studies,^50^ seems to reflect a prolonged selective pressure exerted by the plants on the bacterial populations in the rhizosphere, which was of course absent in the nonplanted controls. Taken together, the analyzed α-diversity indices indicated that the microbial communities associated with P1 (*P. arundinacea*) and P3 (*F. arundinacea*) were the most impacted by the applied treatment and showed the higher number of significantly different indices compared to the related unplanted control (C2). Treatments P1 and P3 displayed a higher diversity of the fungal community, and the former showed this result also for its bacterial community compared to C2. In addition, although both these treatments led to more specialized soil bacterial communities, their members were more evenly distributed compared to the control. Thus, even though we detected a rhizosphere selective effect likely mediated by root-exudate release^51^ in P1 and P3 the higher evenness in the bacterial community could potentially guarantee a better functional stability under stressed and fluctuating environmental conditions.^52^

### 3.3 Effect of Treatments on PCDD/F Depletion

**Figure 1:**
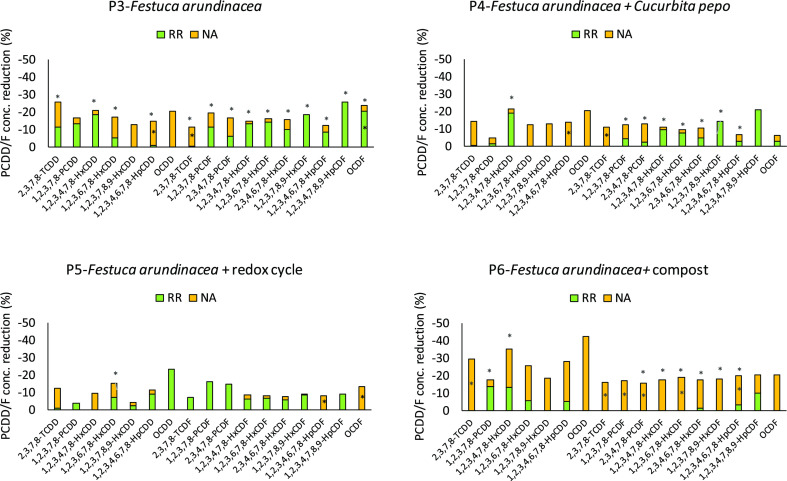
Contribution of NA and RR to the overall PCDD/F concentration reduction at T4. Asterisks indicate statistically significant reduction for NA (when contained in the orange bar), for RR (when contained in the green bar), and overall (when the asterisk is above the bar). (For interpretation of the references to color in this figure legend, the reader is referred to the web version of this article).

PCDD/F concentrations in control and treatment soils were compared to investigate the effect of treatment (plant and soil conditions) on PCDD/F disappearance with time. In general, as previously shown for PCBs^25^ no significant concentration reduction appeared in control and treatment pots after 6 months (T2) from potting, probably due to the short time available to show a significant decrease. As shown for NA (Table S7), RR (Table S9), and OVERALL (Table S11) few exceptions appeared, especially for 2,3,7,8-TCDF, while most of the values oscillate without showing a significant reduction. The most significant results were achieved after 18 months (T4) from potting. Figure 1 shows the contribution of NA and RR to the overall PCDD/F concentration reduction after 18 months for the best treatment (P3-*F. arundinacea*) and the other 3 treatments with the same plant species but different soil conditions for comparison (P4, P5, P6). The results of the other 6 treatments are depicted in Figure S4. Please note that P6 and its control C5 (both added with compost) were normalized to the soil OC to properly compare the results. Moreover, because mineral fertilization was shown not to alter PCDD/F concentration with time (Figure S5), the comparison between controls and treatments was performed considering the fertilized controls (i.e., C2, C3, C4, C5, and C7). Statistically significant reductions between treatments and the relative controls at T4 were not obtained considering RR only (Table S10); this was recorded although we previously detected, at T4, a rhizosphere effect that positively affected the soil hydrolytic activity and overall bacteria abundance, including the population of potential degraders of aromatic compounds.^25^ However, the biostimulation reported in the previous section in most cases was not sufficient to determine a significant enhancement of PCDD/F degradation through RR alone, compared to the NA process (Figures 1 and S4), probably due to the relatively short time of the greenhouse experiment and the extreme persistence of these compounds, as already reported for PCBs.^25^ However, including also NA and comparing the PCDD/F concentration values of the controls at T0 and treatments at T4, a significant reduction could be appreciated (Table S11) probably because of the joint effect of both mechanisms (and their variability). For this reason, the combined effect of NA and RR must be considered to better understand the real effect of plants and soil conditions on PCDD/F depletion.

*F. arundinacea* (P3) could significantly reduce the PCDD/F concentration in the soil of about 11–24% depending on the congeners and the sum of the 17 PCDD/F congeners of about 20%. The highest contribution was mainly due to RR effects (green bars), suggesting that under the cultivation conditions applied in P3, this species may have induced an increased bioavailability of PCDD/Fs to the soil microorganisms because of a particular root exudation pattern and/or may have been more efficient than other plants in stimulating the activity of naturally occurring microbial degraders. *F. arundinacea* cultivated with *C. pepo* ssp pepo (P4) showed significant reductions especially for PCDF (7–13%) and for the sum of the 17 PCDD/F congeners (about 10%), while *F. arundinacea* cultivated under redox conditions (P5) was not efficient in reducing PCDD/F concentrations with the exception of one congener (1,2,3,6,7,8-HxCDD). *F. arundinacea* with compost addition (P6), similarly to P3, could significantly reduce the concentration of a great number of congeners (14–32%); however, the highest contribution was mainly due to NA effects (yellow bars). This probably means that the compost, being a precursor of dissolved and particulate organic carbon in the soil (DOC and POC, respectively), has the effect of enhancing the mobilization of these hydrophobic compounds and/or make them more bioavailable for degradation by the soil microorganisms. Among the other 6 treatments, only P1 (*P. arundinacea*) and P7 (*M. sativa*) showed significant reduction of the sum of the 17 PCDD/F congeners (about 10%) but of a smaller number of congeners, mainly due to the NA process (Figure S4). Although the investigation of the PCDD/F uptake by plant biomass is not the object of the current study it is worth to show that bioaccumulation in roots/shoots of *F. arundinacea* as well as of *C. pepo* ssp pepo was negligible with respect to degradation. PCDD/F concentrations in roots and shoots were about 0.001–1.5% of the initial concentration in the soil on average, depending on the chlorination class. This confirmed that the chemical disappearance cannot be attributed to plant uptake.

### 3.4 HLs of PCDD/Fs

The results of the current experiment were used to calculate the HLs in the soil for the 17 PCDD/F congeners, comparing the concentrations in the controls at the beginning of the experiment and in the treatments at the end of the experiment (overall HL). Table 2 and Figure S6 show the HLs for the best treatment (P3, *F. arundinacea*) and the range of HLs for the other treatments. Full data can be found in Table S13. *F. arundinacea* could significantly reduce (generally with *p* < 0.05) nearly all 17 PCCD/F congeners; only did *p* be <0.1 for 1,2,3,7,8-PCDD and for 1,2,3,7,8,9-HxCDD and OCDD *p* was slightly larger than 0.1. However, some of the other treatments, that is, P1 (*P. arundinacea*), P4 (*F. arundinacea* in consociation with *C. pepo* spp pepo), and P6 (*F. arundinacea* cultivated with the compost) showed significant reduction of a much lower number of congeners (mainly PCDFs).

**Table 2:** PCDD/F HLs in Soil (Years) at 25 °C Obtained from P3 (*F. arundinacea*) and from Other Treatments^a^

	selected species (P3)	other treatments	
PCDD/F congeners	HL (y)	HL min (y)	HL max (y)
2,3,7,8-TCDD	2.54	2.54	4.46*
1,2,3,7,8-PCDD	3.89*	3.71	5.79^+^
1,2,3,4,7,8-HxCDD	3.00	1.77	2.95
1,2,3,6,7,8-HxCDD	3.82	2.45*	4.40
1,2,3,7,8,9-HxCDD	5.55^++^	3.63^+^	8.35^+^
1,2,3,4,6,7,8-HpCDD	4.41	2.23^+^	8.85
OCDD	3.41^++^	3.34^++^	3.68^+^
2,3,7,8-TCDF	5.80	4.31	8.12
1,2,3,7,8-PCDF	3.36	3.36	5.39
2,3,4,7,8-PCDF	3.99	3.99	6.47
1,2,3,4,7,8-HxCDF	4.35	3.60	6.20
1,2,3,6,7,8-HxCDF	3.99	3.44	10.27
2,3,4,6,7,8-HxCDF	4.27	3.67	6.87
1,2,3,7,8,9-HxCDF	4.56	3.55	7.09
1,2,3,4,6,7,8-HpCDF	5.41	3.25	10.04
1,2,3,4,7,8,9-HpCDF	4.98	3.20	9.49*
OCDF	2.63	2.63	6.98*

a When no symbol is present it means that *p* < 0.05; *: *p* < 0.1; +: 0.164 < *p* < 0.242; ++: 0.101 < *p* < 0.108; for more details of the statistical significance please refer to Table S15 in the Supporting Information.

HLs ranged from 2.5 to 5.8 years considering *F. arundinacea* and from 1.8 to 10.3 considering all the treatments of the experiment and they did not show a linear correlation with congener hydrophobicity as previously reported by other authors.^53,54^ The calculation of HLs was performed for PCDD/F classes in P3 treatment only. It was confirmed that HLs were about 4 years for hexa- and hepta-PCDDs and 5 years for hexa- and hepta-PCDFs. For tetra- and penta-PCDD/Fs, a statistically significant reduction could not be appreciated.

### 3.5 Bound Residue Formation Versus Loss Mechanisms

When evaluating the potential for biodegradation it is important to quantify the amount of bound residues, also called nonextractable residues (NERs).^55−57^ Some authors^53^ suggested that the gradual conversion of PCDD/Fs to NER could be one reason for their disappearance, independently of the chlorine number, in contaminated sewage sludge amended soil over a period of ∼20 years. However, the formation of bound residues usually derives by the long contact times of persistent chemicals (such as PCDD/Fs) with soil particles and organic matter. In fact, in the case of Brescia soils, we^22^ previously showed that the contamination by PCBs and PCDD/Fs was related to the long-time industrial production of the Caffaro plant. This means that if bound residues were formed, they probably developed in the past 80 years of cultivation, before the start of the RR experiments. It is therefore unlikely that significant amounts of new bound residues could be formed in the following 18 months of RR. It must also be considered that the extraction method employed (pressurized liquid extraction using toluene as the extraction solvent) is known to be an exhaustive extraction method.^58−60^ This method is even more efficient than the one used by Seike and coauthors^54^ who, using toluene with a Soxhlet apparatus, showed that the contribution of the bound residue formation to PCDD/Fs disappearance in the paddy field soil was not significant (i.e., less than 10% in ∼20 years). This means that the extraction method employed could reveal a change PCDD/F concentrations with time, and this cannot be attributable to the formation of bound residues but to a loss process such as biodegradation, volatilization, vertical infiltration, or so forth. While the relevance and the true responsibility among these loss processes will be evaluated in the following section, it could be worthwhile to investigate whether the PCDD/Fs measured at T4 are to be considered fully bioaccessible and therefore available for biodegradation processes. In fact, it was reported^16,61^ that “NER are the chemical species that are unextracted by methods which do not significantly change the chemical nature of these residue and whose formation reduces the bioaccessibility and bioavailability significantly”. This means that the NER formation will reduce the bioaccessible concentration in soil pore water. In order to verify whether bound residues were responsible for such inaccessibility of PCDD/Fs, we used roots as a “sensor” for the bioaccessible fraction in water of PCDD/Fs. While the complete set of data for all the species will be presented in separate paper, the root concentration data measured at T4 for P3 (tall fescue) were used to back calculate the concentrations of PCDD/Fs in soil water (*C*_pw(2)_) needed to reach the concentrations in roots predicted by a root uptake equation^43^ developed for a comparable species (barley). Additionally (see Section 2.6), the concentration in soil pore water (*C*_pw(1)_) was calculated starting from measured soil concentrations and the soil water partition coefficient (*K*_d_). The results of the comparison of the two estimated pore water concentrations are shown in Table S14. The values of pore water concentrations calculated from root concentrations are generally comparable to or higher than those calculated from soil concentrations, showing that no relevant bound residue fractions of PCDD/Fs could be detected. This means that, although some uncertainty may arise from the estimation, the pore water concentrations for PCDD/Fs are in equilibrium with the concentrations measured in soil and are therefore to be considered bioaccessible.

Additionally, the HL data of Table 2 show little variability among the different chemicals, in other terms the influence of the structural properties of PCDD/Fs (e.g., the number of chlorine) is not apparent. This is also shown by most of the literature available (see section below) and can also be explained considering different aspects. First of all, given the prolonged contamination of the experimental soil by PCDD/Fs, it could be expected that the autochthonous microflora has adapted to degrade these chemicals, and it is strictly related to the bioaccessible fraction in water. When the release of these chemicals in pore water is enhanced (due, e.g., to the degradation of OC and the release of the PCDD/Fs therein associated), PCDD/F degradation could start at a faster pace, being the OC carbon mineralization the limiting step.^16^ Second, during mineralization also the production of DOC has the effect of spreading these immobile chemicals in soil and making them available to soil microorganisms, as recently suggested.^16,29^ This has the effect of synchronizing chemical degradation to the OC cycle.^62,63^ This is further corroborated by analyzing the results of the study on the soil hydrolytic activity in the control and treatment pots, through the measurement of the fluorescein diacetate hydrolysis test.^25^ The soil hydrolytic activity increased with time in all the soils subjected to plant biostimulation (i.e., in planted pots but not in unplanted pots). Considering that soil hydrolytic activity can be seen as a proxy for biodegradation of the soil organic matter, an increase in hydrolytic activity in contaminated soil could be followed by the biodegradation of contaminants, as reported above.^25^ Additionally, we have also shown that the cultivation of plants in the contaminated soil has the effect of stimulating the growth of microorganisms in soil, as shown by the increased diversity, evenness, and richness indices as reported in Section 3.2.

### 3.6 Literature Comparison

#### 3.6.1 Natural Attenuation

Few data about PCDD/F degradation HLs in the soil compartment are available in the literature. Some of the data sets^64−66^ are generally used as input parameters in models to predict the environmental fate of this type of organic contaminants.^22,67−71^ However, the data in these data sets could differ up to an order of magnitude from the corresponding data obtained in the current study (Table S15). In addition, the study of Sinkkonen and Paasivirta^66^ reported HLs for soils which were derived from the data obtained from the Baltic Sea Sediment^72^ because the soil HLs were not available. However, these HLs were considerably higher than those of Mackay et al.,^64,65^ who assigned PCDD/Fs to different HL classes (e.g., 8 and 9) which represent highly persistent compounds (1–11 years). The sensitivity analysis of different environmental fate models^30,73,74^ highlighted that HLs in the soil are among the most important parameters in determining the concentration of persistent organic contaminants in the soil. This means that the selection of the correct input value is a crucial step to obtain reliable results. Other data available for the soil compartment refer to both laboratory and field studies (Table S15). One study^75^ showed that the concentration of PCDD/Fs in contaminated soil located around two industrial plants in South Germany did not change significantly over a 2 year-period and a significant change in the homologue patterns was not appreciated. Similarly, another study^76^ showed that the TCDD concentration in a spiked soil did not significantly change during a one-year column experiment. On the contrary McLachlan et al.,^53^ measured PCDD/F concentration reduction in a soil amended with contaminated sludge over a 20 year period; because no shift in the congener pattern was observed, they ascribed the concentration reduction to the phenomenon of aging, which reduced the extractable amount of PCDD/Fs. These data are quite in line with those reported for a Japanese paddy field;^54^ however, the disappearance was instead attributed^54^ to other processes such as volatilization, photolysis, biodegradation, infiltration, and runoff as possible causes for the decline of PCDD/Fs from paddy soils. Other authors^77^ showed, through a 14-months microcosm experiment with a spiked soil, that some penta-, hexa-, and hepta-PCDD/F concentrations can decrease up to 56% with respect to the control (autoclaved microcosm) and the HLs obtained ranged between 2.5 and 3.5 years. Similarly, HL values of about 1 year for 2,3,7,8-TCDD during a 1 year laboratory experiment with a spiked soil were reported.^78^ During a degradation test in the laboratory with OCDD spiked soil a HL of 1.7 years was obtained.^79^ Other authors estimated a HL of 14 years for OCDD.^80^ Finally^81^ showed that some hepta-PCDF and OCDF concentrations can decrease up to 99% in 12 weeks during a laboratory experiment with aged soil. All these data refer to NA, that is, chemical disappearance from the soil because of volatilization, infiltration, runoff, and degradation including baseline biodegradation by autochthonous microorganisms. The comparison of HLs obtained in the current experiment with those present in the NA literature shows that the data presented in this study are generally smaller, indicating the importance of the additional effect of RR, that is, the plant selection and root-microbe interactions, in the degradation of these compounds.

#### 3.6.2 Bioaugmentation Experiments

Many microorganisms were isolated from different environmental matrices and identified as PCDD/F degraders.^1,82,83^ Both bacteria and fungi were used in bioaugmentation experiments, with both spiked and aged PCDD/F contaminated soil or growth medium, resulting in shorter HLs (in the order of days/months) than those presented in this work (Table S16). However, in these studies, the selected microorganisms are used in a simplified system and under the best environmental conditions for degradation (e.g., nutrients, temperature, and oxygen), maximizing contaminant bioavailability, and concentration levels (e.g., spiked soil/medium). As outlined in a recent paper of our group,^16^ caution must be taken in translating these results to the real-field scale where a number of additional factors may influence HLs such as the spatial variability of soil properties and chemical contamination, the contaminant bioavailability to microorganisms, the soil water and nutrient content, as well as proper temperature conditions.

#### 3.6.3 RR Studies

Although many studies investigated the degradative potential of different microorganisms for PCDD/F, few works focused on plant–microbe interactions during the RR process (Table S17). For example, some authors^21^ measured the degradation of dibenzofuran in a spiked soil cultivated for 2 months with three grasses (*Cynodon dactylon*, *Agrostis palustris*, and *Zoysia japonica*) and a leguminous (*Trifolium repens*); HLs ranged from 36 to 106 days depending on the plant species. The same authors^20^ later used *T. repens* to degrade a mixture of PCDD/Fs (DF, 2,8-DCDF, 2,4,8-TCDF, DD, 1-CDD, 2,7-DCDD, 1,2,4-TCDD, and 1,2,3,4-TCDD) in a spiked soil over a 12 week period; however, just few congeners were significantly reduced (HLs ranged from 122 to 231 days). Similarly, other authors^17^ investigated the effects of mycorrhizal fungi on association with *M. sativa* and *F. arundinacea* on the removal of PCDD/F from a historically polluted soil after 24 weeks of culture in microcosms. Although significant decreases ranging from 22 to 35% were observed in planted microcosms for total PCDD/F concentrations, no data were available to calculate the HLs for single PCDD/F congeners similarly to a companion paper.^47^ As shown in Table S17, literature data could not be properly compared to the results of the current experiment because of the differences in the experimental design including the type of the contamination (spiked vs aged), the experiment time (84 days vs 553 days), and the degree of chlorination of the investigate congeners (1–4 chlorines vs 4–8 chlorines).

### 3.7 Environmental Factors Influencing HLs

**Figure 2:**
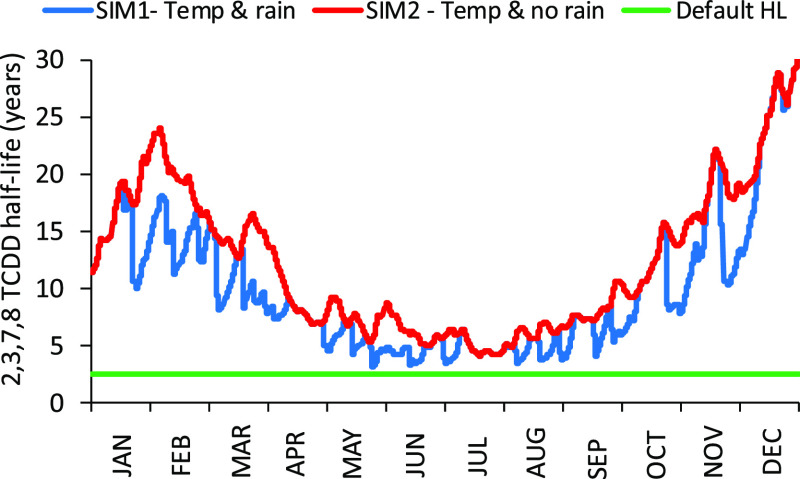
Temperature and water influence on 2,3,7,8-TCDD degradation HL in the soil.

Temperature and water availability are among the most important environmental factors that drive contaminant degradation in the soil.^84^ It is important to highlight that the current experiment was performed in a greenhouse that was not thermostatically controlled; therefore, air temperatures were roughly reflecting real seasonal climatic conditions.^25^ This temperature trend allows to obtain more relevant results for field conditions than those obtained in climatically controlled chambers, which may represent unrealistic degradation rates for the entire year. Additionally, irrigation was carefully adjusted to guarantee a comparable water content (maintained at about 30% of the soil total volume) in both control and treatment pots and avoid the occurrence of saturated and anoxic conditions for the unplanted controls. This means that to obtain similar results in field conditions, irrigation is needed, especially during dry seasons. The SoilPlusVeg model was run for a year using default HL at 25 °C for P3 (Table 2) and using an hourly temperature and rainfall data for 2,3,7,8 TCDD. Because SoilPlusVeg can recalculate the HLs considering each of the hourly conditions following a common approach,^84^ it is important to evaluate how the expected biodegradation rates would change accordingly. Figure 2 shows the seasonal variability of degradation HL for 2,3,7,8-TCDD considering simulation 1 (SIM1—temperature and rain) and simulation 2 (SIM2—temperature and no rain); the default HL value (at 25 °C) derived from the P3 result (i.e., 2.54 year) is also reported. Degradation HLs could vary up to one order of magnitude during the year because of the combined effect of temperature and water (SIM1-temp and rain); when rain is ignored in the simulation scenario (SIM2-temp), degradation HLs are up to a factor of 2 higher.

### 3.8 Influence of Other Loss Processes on Half-Life

**Figure 3:**
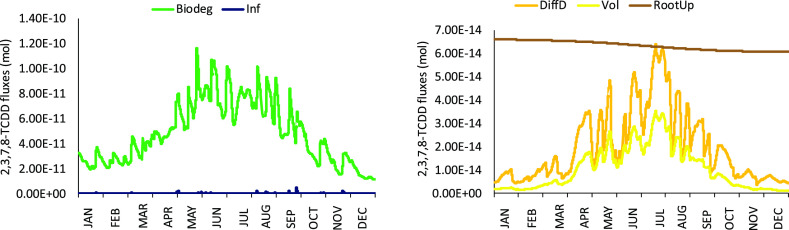
Temporal variability of 2,3,7,8-TCDD losses from soil, i.e., biodegradation (Biodeg), infiltration (Inf), diffusion down (DiffD), volatilization (Vol), and root uptake (RootUp).

The HLs obtained in the current experiment (Table 2) could be considered as “overall HLs” and include NA and RR as explained above, being the results of biological degradation by biostimulated autochthonous microorganisms. However, because it is calculated as disappearance time (time needed for the loss of 50% of the compound), it could possibly include other loss processes than biodegradation, such as volatilization, infiltration, diffusion downward, and root uptake. In order to rule out the importance of each process on PCDD/F fate in the soil, some simulations were performed for three representative PCCD/Fs of increasing/decreasing values of physico-chemical properties (i.e, 2,3,7,8-TCDD, 1,2,3,4,7,8-HxCDF and OCDD) (see Table S2).·As shown in Table 3 and Figure 3, biodegradation represents the most important loss process in the soil compartment for all simulated PCDD/F congeners. Diffusion downwards, volatilization and root uptake could be considered negligible, never exceeding 1% of the overall losses; infiltration of truly dissolved chemical and associated to DOC could be on average negligible too but, in few moments of the year when specific conditions can occur (i.e., when infiltration water is maximized due to heavy rainfall/irrigation and DOC concentrations are higher), it could represent between 2 and 10% of the overall losses from soil. For the most hydrophobic compounds (such as OCDD and OCDF) the influence of moving POC in soil can slightly enhance the losses through this mechanism,^85,86^ as well as the effect of plants on the soil structure as revealed in a recent soil column experiment.^87^ As reported in 3.3 PCDD/F concentration reductions varied from 6 to 32% on average during the whole experiment (18 months). This means that the obtained HLs could be mainly ascribed to the naturally occurring microbial biodegradation process, in some cases enhanced by plant biostimulation (e.g., P3) or compost amendment (e.g., P6 and C5). This is also confirmed by the fact that (1) water infiltration on pot was minimized maintaining the water content at about the 30% of soil total volume and (2) the PCDD/F concentrations measured in the above ground biomass of control C6 (planted uncontaminated soil) were negligible, indicating low volatilization of these chemicals from the contaminated soil pots.

**Table 3:** PCDD/F Loss Processes in the Soil Compartment^a^

process	Biodeg	Inf	DiffD	Vol	RootUp
loss fluxes (%)					
2,3,7,8-TCDD					
mean	99.61	0.16	0.04	0.02	0.18
min	91.55	0.00	0.01	0.00	0.06
max	99.90	8.16	0.07	0.04	0.54
1,2,3,4,7,8-HxCDF					
mean	99.59	0.19	0.02	0.01	0.19
min	89.76	0.00	0.01	0.00	0.06
max	99.92	10.04	0.04	0.02	0.58
OCDD					
mean	99.95	0.04	0.00	0.00	0.01
min	97.58	0.00	0.00	0.00	0.00
max	100.00	2.42	0.00	0.00	0.03

a Biodeg: biodegradation; Inf: infiltration; DiffD: diffusion downwards; vol: volatilization; RootUp: root uptake.

### 3.9 Importance of the HL Data set in Fate Modelling and Remediation

The data produced in this study derive from an accurate selection of the plant species which could be relevant in common agricultural situations and/or characterizing a permanent grassland. The different soil treatments were chosen after an extensive literature search on PCB phytoremediation^15,16^ considering the main contaminants and environmental characteristics of the SIN. PCDD/F concentrations were measured in controls and treatments at different intervals during the experiment to investigate the role of NA and of RR processes on PCDD/F depletion over time: these results were used to derive a range of min/max values and typical values of HLs for all the TEF holding PCDD/Fs. Such values can be efficiently used to update the simulations on the long range movement of these chemicals, as required by the Stockholm convention on POPs^88^ or better predict the temporal transfer in food chain models^67,89^ in order to reduce the risk for ecosystems and human health. Additionally, the data obtained can be used as guidance to direct field-based bioremediation studies in contaminated sites.

## Supporting Information Available

Additional information regarding the experimental design, analytical methods, as well as simulation scenario and detailed results (PDF)
