# Supplementary material for: New Data Set of Polychlorinated Dibenzo-p-dioxin and Dibenzofuran Half-Lives: Natural Attenuation and Rhizoremediation Using Several Common Plant Species in a Weathered Contaminated Soil
Source: Environ Sci Technol. 2020 Jul 20;54(16):10000–11. doi: 10.1021/acs.est.0c01857 (PMC8009521; doi:10.1021/acs.est.0c01857)
Supplement: Supplementary file 1 — es0c01857_si_001.pdf [file es0c01857_si_001.pdf]

**SUPPORTING INFORMATION FOR**

# A new dataset of Polychlorinated Dibenzo-p-Dioxin and Dibenzofuran half-lives: natural attenuation and rhizoremediation using several common plant species in a weathered contaminated soil

Elisa Terzaghi[a], Lorenzo Vergani[b], Francesca Mapelli[b], Sara Borin[b], Giuseppe Raspa[c], Elisabetta Zanardini[a], Cristiana Morosini[a], Simone Anelli[d], Paolo Nastasio[d], Vanna Maria Sale[d], Stefano Armiraglio[e], Antonio Di Guardo[a*]

[a]DiSAT, University of Insubria, Via Valleggio 11, Como, Italy
[b]DeFENS, University of Milan, Via Celoria 2, Milan, Italy
[c]DICMA, Sapienza University of Rome, Via Eudossiana 18, Rome, Italy
[d]ERSAF, Via Pola 12, Milan, Italy
[e]Municipality of Brescia - Museum of Natural Sciences, Via Ozanam 4, Brescia, Italy

Contains 24 pages, 6 figures and 17 tables

**TABLE OF CONTENTS**

S1 – MATERIAL AND METHODS ..... S2
PCDD/F ANALYSIS ..... S2
SIMULATION SCENARIOS ..... S4
S2 – RESULTS AND DISCUSSION ..... S5
PCDD/F INITIAL CONCENTRATION IN SOIL ..... S5
IMPACT OF THE TREATMENTS ON THE ALPHA-DIVERSITY OF THE MICROBIAL COMMUNITIES ..... S8
NATURAL ATTENUATION EFFECT ..... S10
RHIZOREMEDIATION EFFECT ..... S11
OVERALL EFFECT ..... S12
CONTROL COMPARISON: UNFERTILIZED vs. FERTILIZED ..... S14
HALF-LIVES ..... S15
BIODEGRADATION vs. BOUND RESIDUE FORMATION ..... S17
REFERENCES ..... S22

[*] Corresponding author e-mail: antonio.diguardo@uninsubria.it

**S1 – MATERIAL AND METHODS**

**PCDD/F ANALYSIS**

10 g or soil were mixed with Diatomaceus Earth and the following $^{13}C$ labeled internal standards were added: 2,3,7,8 TCDD, 1,2,3,7,8 PeCDD, 1,2,3,4,7,8 HxCDD, 1,2,3,6,7,8 HxCDD, 1,2,3,4,6,7,8 HpCDD OCDD, 2,3,7,8 TCDF, 1,2,3,7,8 PeCDF, 2,3,4,7,8 PeCDF, 1,2,3,4,7,8 HxCDF, 1,2,3,6,7,8 HxCDF, 2,3,4,6,7,8 HxCDF, 1,2,3,7,8,9 HxCDF, 1,2,3,4,6,7,8 HpCDF, 1,2,3,4,7,8,9 HpCDF. Samples were extracted with an Accelerated Solvent Extractor Thermo Scientific DIONEX ASE 350, using toluene and acetone according to this method: temperature, 150°C; heat, 7 min; static time, 7 min; cycles, 2; rinse volume, 60%; purge, 180 s; cell type, SST. Before purification the following clean up standard was added: 2,3,7,8-TCDD $^{37}Cl$. The soil and biomass extracts were purified using silica/alumina FMS columns and hexane and dichloromethane as solvents with the FMS POWER-PREP system. Samples were concentrated under $N_2$ flow and analyzed in HRGC/HRMS (Thermo Scientific TRACE GC ULTRA coupled with Thermo Scientific DFS MS) after $^{13}C$ labeled internal standards addition (1,2,3,4-TCDD and 1,2,3,7,8,9-HxCDD), using a RTX Dioxin 2 column (60m, 0.25 mm ID, 0.25 mm film thickness) with He as carrier gas at a constant flow of 1 ml $min^{-1}$. The GC operating conditions were as follows: injector (splitless) temperature 260°C, interface temperature 270°C, initial temperature 130°C, initial time 1 min. The GC temperature program was 130-200 °C at 40 °C $min^{-1}$, 200-235 °C at 3°C $min^{-1}$, and final time 25 min. Total run time was The monitored masses were, M+ and M+2 (for tetra and penta Cl classes) and M+2 and M+4 ( for hexa, hepta, octa Cl classes). 17 congeners and PCDD/F classes were quantified (**Table S1**).

**Table S1 - Measured PCDD/F congeners**

| | |
|---|---|
| **PCDDs** | 2,3,7,8-TCDD |
| | 1,2,3,7,8-PCDD |
| | 1,2,3,4,7,8-HxCDD |
| | 1,2,3,6,7,8-HxCDD |
| | 1,2,3,7,8,9-HxCDD |
| | 1,2,3,4,6,7,8-HpCDD |
| | OCDD |
| **PCDFs** | 2,3,7,8-TCDF |
| | 1,2,3,7,8-PCDF |
| | 2,3,4,7,8-PCDF |
| | 1,2,3,4,7,8-HxCDF |
| | 1,2,3,6,7,8-HxCDF |
| | 2,3,4,6,7,8-HxCDF |
| | 1,2,3,7,8,9-HxCDF |
| | 1,2,3,4,6,7,8-HpCDF |
| | 1,2,3,4,7,8,9-HpCDF |
| | OCDF |

## SIMULATION SCENARIOS

### SELECTED CHEMICALS

**Table S2 - Physico-chemical properties of the simulated chemicals**

| | 2,3,7,8-TCDD | 1,2,3,4,7,8-HxCDF | OCDD |
|---|---|---|---|
| **MW (g mol$^{-1}$)** | 322 | 375 | 460 |
| **VP (Pa)** | 0.0000002 | 0.000000032 | 0.00000000011 |
| **WS (g m$^{-3}$)** | 0.0000195 | 0.00000825 | 0.0000000740 |
| **Log $K_{OW}$** | 6.8 | 7 | 8.20 |
| **HL air (days)** | 7.08 | 22.9 | 22.9 |
| **HL soil (days)** | 972 (2.54 years) | 1589 (4.35 years) | 1244 (3.41 years) |

**NOTE:** MW, VP, WS, Log $K_{OW}$ and HL air were taken from [1], while for HL soil the results of the current experiment for P3 treatment (*Festuca arundinacea*) were used

### AIR, VEGETATION, SOIL COMPARTMENT PARAMETERIZATION

A 40 cm (5 layers of 8 cm each) loamy sand soil characterized by 1.7% organic carbon (OC) and an average DOC concentration in soil water of ~15 mg L$^{-1}$ was simulated. The model domain was set to 1 ha to reproduce an agricultural field of *Festuca arundinacea*. A below ground biomass of 1.932 kg m$^{-2}$ and an above ground biomass of 0.9 kg m$^{-2}$ were assumed [2], while Specific Leaf Area (SLA) and Leaf Area Index (LAI) were set to of 10 m$^2$ kg$^{-1}$ and a of 9 m$^2$ m$^{-2}$ respectively [3]. A well-mixed and homogeneous soil was simulated considering a fixed initial concentration for each soil layer at the beginning of the simulation. The initial concentration was set equal to the concentrations in control pots at the beginning of the experiment (**Table S3**). The air compartment structure and surface meteorological parameters (temperature, rainfall and solar radiation) were parameterized as reported in [4]. A clean air compartment was considered at the beginning of the simulations (no background concentration and emission to air), while PM10 background concentration was set to 30 µg m$^{-3}$.

**Table S3 - PCDD/F emissions and initial concentrations in soil**

| | 2,3,7,8-TCDD | OCDD | 1,2,3,4,7,8-HxCDF |
|---|---|---|---|
| **Emission (mol)** | 0.00000469 | 0.00381 | 0.00164 |
| **Initial concentration (pg g$^{-1}$ dw)** | 1 | 154 | 542 |

## S2 – RESULTS AND DISCUSSION

### PCDD/F INITIAL CONCENTRATION IN SOIL

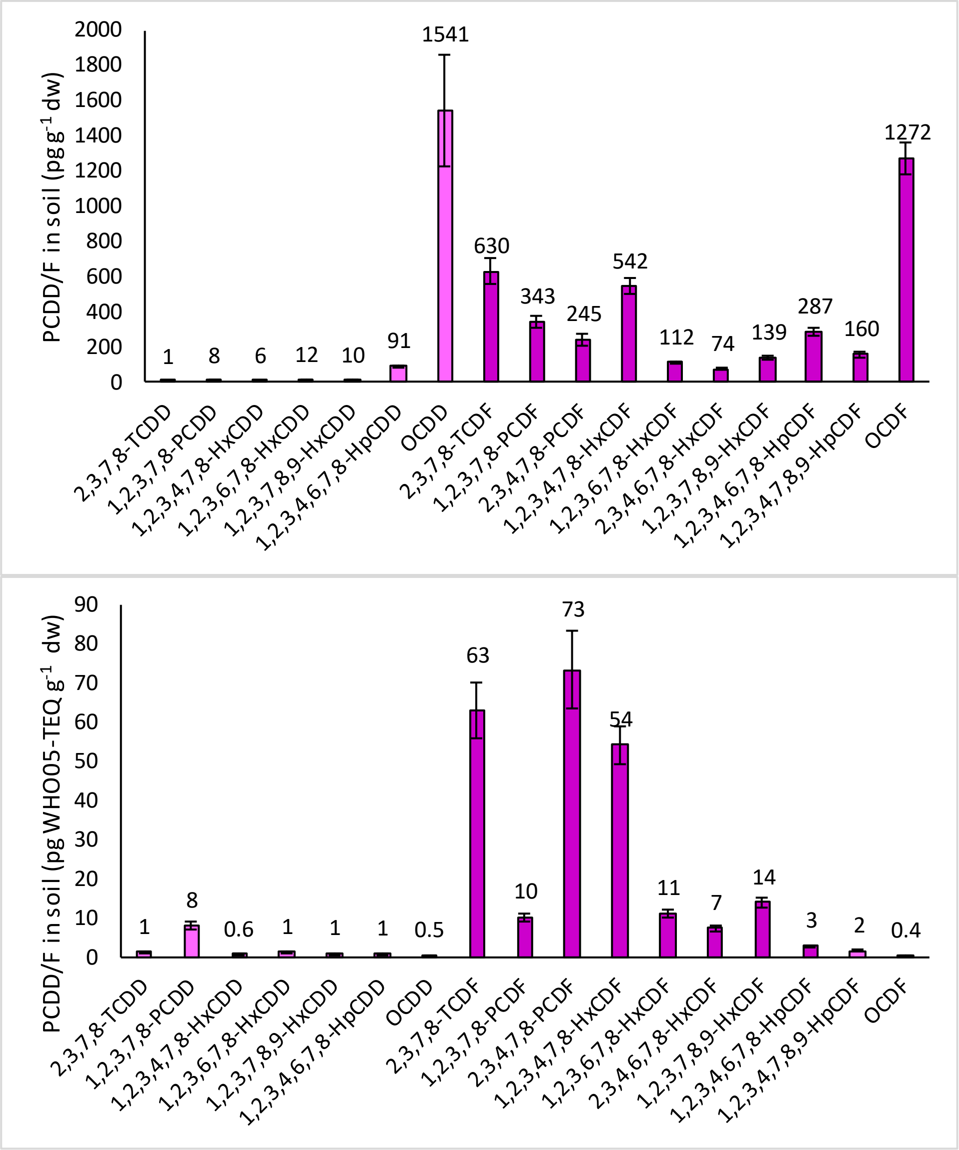


**Figure S1. PCDD/F initial concentration in soil at T0 (C1-4).** Natural concentrations are represented on the top, while WHO05-TEQ are represented on the bottom. PCDDs are shown in pink while PCDFs are shown in violet. Bars represent standard deviation of 12 replicates

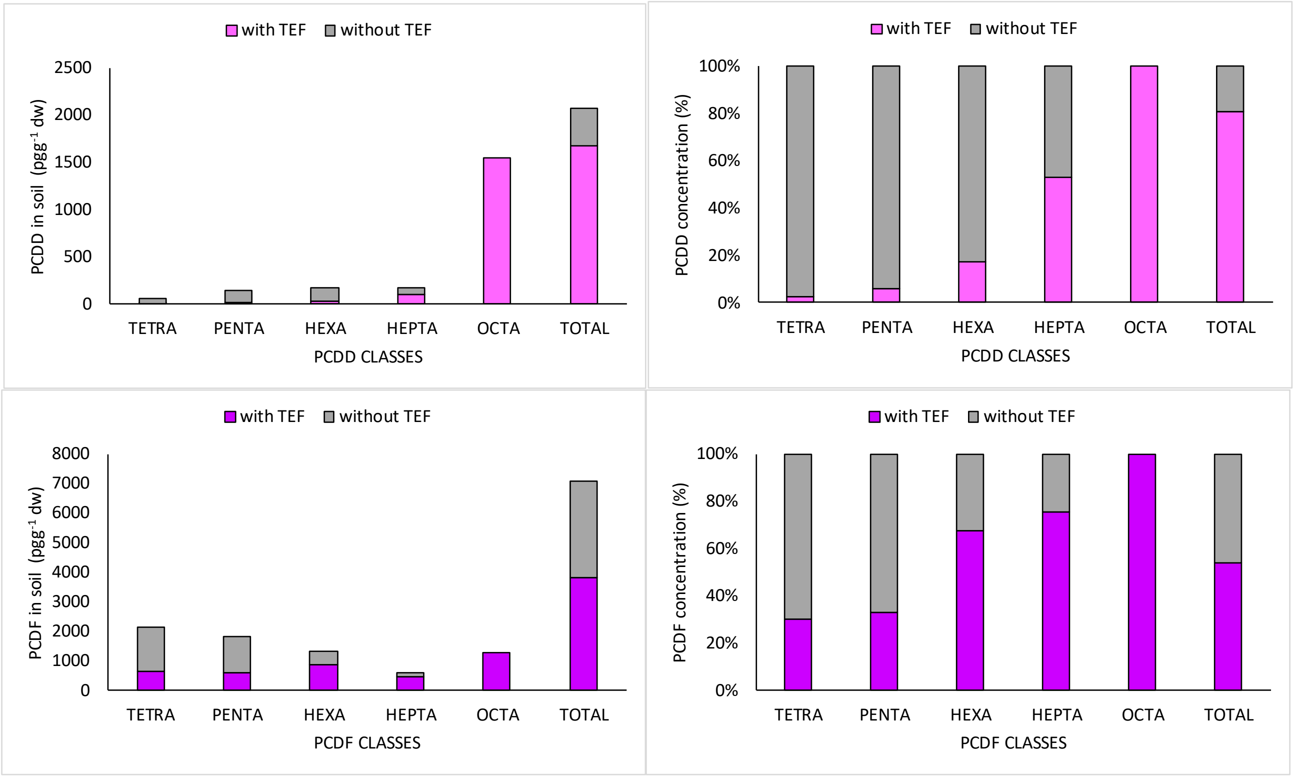


**Figure S2. PCDD/F classes initial average concentration in soil at T0 (C1-4).** PCDDs with TEF are shown in pink while PCDFs with TEF are shown in violet; congeners without TEF are shown in grey

**Table S4 - PCDD/F concentrations in control pots at T0**

| | PCDD/F concentrations in soil pg $g^{-1}$ dw | | | | | |
|---|---|---|---|---|---|---|
| | PCDD | | PCDF | | PCDD/F | |
| | Average | Stdev | Average | Stdev | Average | Stdev |
| **C1-4-T0** | 1670 | 309 | 3802 | 310 | 5472 | 497 |
| **C5-T0** | 1802 | 974 | 3333 | 94 | 5135 | 890 |
| **C6-T0** | 19 | 16 | 16 | 4 | 35 | 6 |
| **C7-T0** | 1479 | 204 | 3744 | 61 | 5223 | 262 |

**Table S5 – Single PCDD/F congener comparison between controls (p-values)**

| | p-values | | |
|---|---|---|---|
| | **C1-4-T0 vs C5-T0** | **C1-4-T0 vs C7-T0** | **C5-T0 vs C7-T0** |
| **2,3,7,8-TCDD** | 0.07 | 0.15 | 0.72 |
| **1,2,3,7,8-PCDD** | 0.14 | 0.30 | 0.45 |
| **1,2,3,4,7,8-HxCDD** | 0.14 | 0.06 | 0.56 |
| **1,2,3,6,7,8-HxCDD** | 0.44 | 0.35 | 0.92 |
| **1,2,3,7,8,9-HxCDD** | 0.36 | 0.80 | 0.59 |
| **1,2,3,4,6,7,8-HpCDD** | 0.86 | 0.64 | 0.77 |
| **OCDD** | 0.67 | 0.36 | 0.60 |
| **2,3,7,8-TCDF** | 0.06 | 0.80 | **0.0003** |
| **1,2,3,7,8-PCDF** | **0.02** | 0.46 | **0.0004** |
| **2,3,4,7,8-PCDF** | 0.09 | 0.58 | **0.0050** |
| **1,2,3,4,7,8-HxCDF** | **0.03** | 0.74 | **0.03** |
| **1,2,3,6,7,8-HxCDF** | **0.02** | 0.88 | **0.01** |
| **2,3,4,6,7,8-HxCDF** | **0.05** | 0.80 | **0.01** |
| **1,2,3,7,8,9-HxCDF** | **0.04** | 0.84 | **0.05** |
| **1,2,3,4,6,7,8-HpCDF** | 0.07 | 0.75 | **0.0019** |
| **1,2,3,4,7,8,9-HpCDF** | 0.09 | 0.79 | 0.09 |
| **OCDF** | **0.02** | 0.86 | **0.03** |
| **SUM** | 0.39 | 0.44 | 0.88 |

**NOTE:** Values in bold represent statistically significant differences in concentrations (**α**=0.05)

**IMPACT OF THE TREATMENTS ON THE ALPHA-DIVERSITY OF THE MICROBIAL COMMUNITIES**

**Table S6. Student t-test comparison of the bacterial (A) and fungal (B) community structure in the different treatment and control theses at T4.** The table includes the p-value calculated comparing the ecological index values for each treatment and the correspondent control. Statistically significant differences are reported in bold. The considered ecological indices are Richness, Diversity (expressed as Shannon index) and Evenness, and their values are reported in Supplementary Figure S3.

| (A) Bacterial community | | | |
|---|---|---|---|
| | Richness | Diversity | Evenness |
| P1 vs C2 | **0.029** | **0.006** | **0.003** |
| P3 vs C2 | **0.002** | 0.105 | **0.003** |
| P4 vs C2 | **0.001** | 0.935 | 0.107 |
| P7 vs C2 | **0.028** | 0.935 | 0.574 |
| P9 vs C2 | 0.079 | **0.003** | **0.007** |
| P2 vs C3 | **0.031** | 0.255 | 0.072 |
| P5 vs C3 | **0.002** | 0.533 | 0.081 |
| P10 vs C4 | **0.037** | 0.200 | 0.398 |
| P6 vs C5 | **0.027** | 0.690 | 0.797 |
| P8 vs C7 | 0.392 | **0.046** | **0.018** |
| (B) Fungal community | | | |
| | Richness | Diversity | Evenness |
| P1 vs C2 | 0.383 | **0.011** | **0.027** |
| P3 vs C2 | 0.385 | **0.019** | 0.058 |
| P4 vs C2 | 0.568 | **0.049** | 0.118 |
| P7 vs C2 | 0.769 | **0.043** | 0.416 |
| P9 vs C2 | 0.394 | 0.686 | 0.698 |
| P2 vs C3 | 0.973 | 0.699 | 0.386 |
| P5 vs C3 | 0.246 | 0.603 | 0.342 |
| P10 vs C4 | 0.892 | 0.859 | 0.371 |
| P6 vs C5 | 0.155 | 0.149 | 0.401 |
| P8 vs C7 | 0.926 | 0.552 | 0.306 |

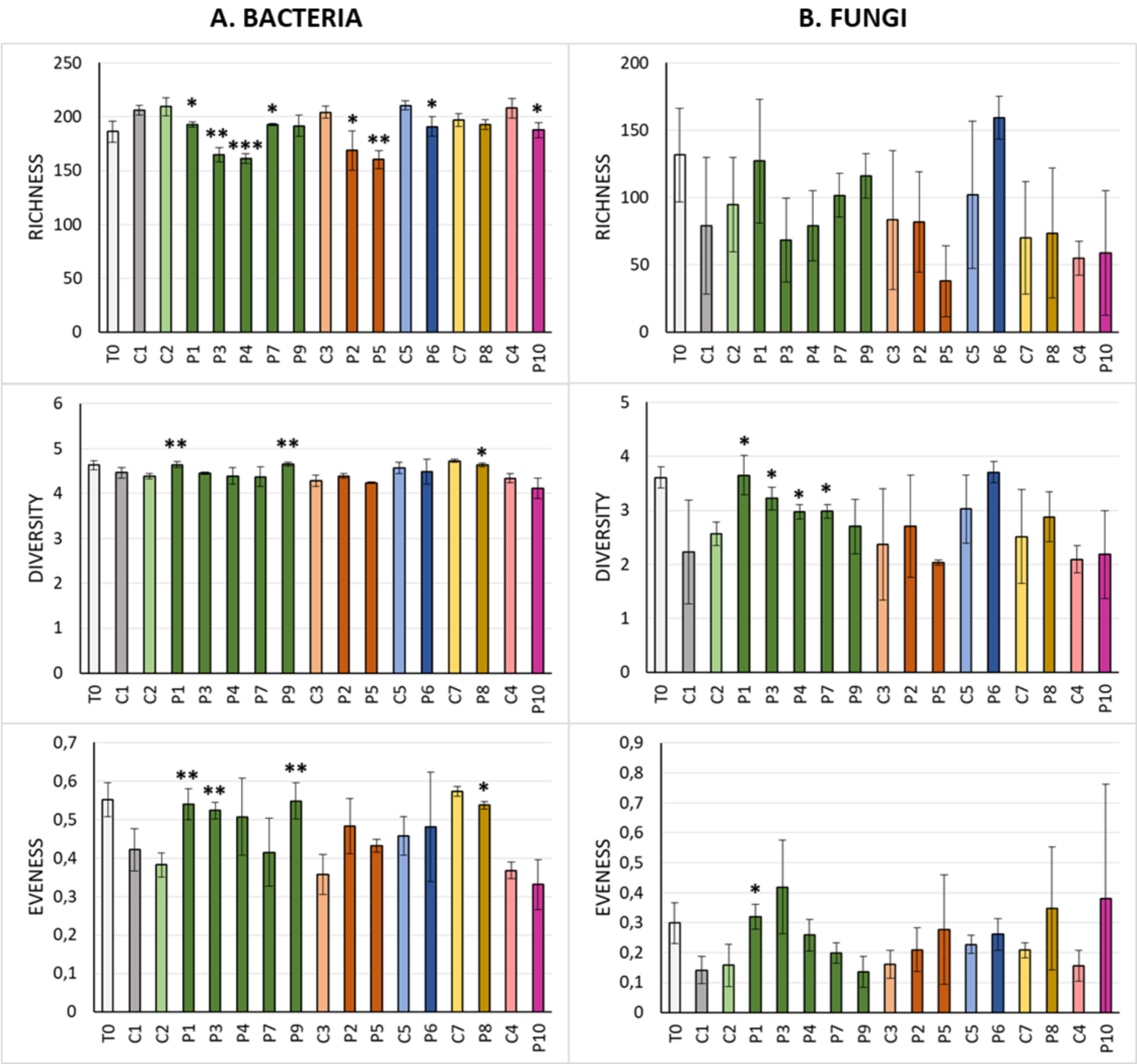


**Figure S3. Richness, Diversity and Evenness of the bacterial and fungal communities at T4.** Diversity indices of the bacteria communities are represented on the left (A) while those of the fungal communities are represented on the right (B). Different treatments and the correspondent control at T4 are indicated by the same color in the chart. The white bar represents the data of the bacterial (A) and fungal (B) communities dwelling the soil before treatment (T0). Asterisks indicate the significant differences between treatments and controls at T4 according to the Student-t test (*$p < 0.05$, **$p < 0.01$, ***$p < 0.001$).

## NATURAL ATTENUATION EFFECT

### Table S7 - Natural attenuation effect: C-T0 vs C-T2 (significant reduction in grey, ns: not significant, s: significant)

| | PCDD/F concentration reduction (%): C-T0 vs C-T2 ($NA_{T2}$) | | | | | | | | | |
|---|---|---|---|---|---|---|---|---|---|---|
| | C1-4-T0 vs C2-T2 | | C1-4-T0 vs C3-T2 | | C1-4-T0 vs C4-T2 | | C5-T0 vs C5-T2 | | C7-T0 vs C7-T2 | |
| **2,3,7,8-TCDD** | ns | -6.38 | ns | 32.17 | ns | 10.39 | ns | 34.45 | ns | 13.92 |
| **1,2,3,7,8-PCDD** | ns | 1.88 | ns | 39.69 | ns | -6.72 | ns | 16.92 | ns | 17.54 |
| **1,2,3,4,7,8-HxCDD** | ns | -6.51 | ns | 36.60 | ns | -7.13 | ns | 4.53 | ns | 12.97 |
| **1,2,3,6,7,8-HxCDD** | ns | -4.02 | ns | 67.42 | ns | 0.12 | ns | 9.62 | ns | 5.93 |
| **1,2,3,7,8,9-HxCDD** | ns | 11.00 | ns | 79.92 | ns | 9.22 | ns | 14.57 | ns | 13.55 |
| **1,2,3,4,6,7,8-HpCDD** | ns | -5.59 | ns | 41.86 | ns | -0.79 | ns | -3.89 | ns | 8.27 |
| **OCDD** | ns | 5.58 | ns | 19.93 | ns | 7.02 | ns | 12.56 | ns | 180.36 |
| **2,3,7,8-TCDF** | s | -19.63 | s | -13.90 | s | -13.75 | s | -8.04 | s | -10.76 |
| **1,2,3,7,8-PCDF** | ns | 1.68 | ns | 7.64 | ns | 6.94 | ns | 13.90 | ns | 9.11 |
| **2,3,4,7,8-PCDF** | ns | 10.16 | ns | 16.06 | ns | 13.55 | ns | 24.03 | ns | 16.05 |
| **1,2,3,4,7,8-HxCDF** | ns | -0.10 | ns | 8.35 | ns | 1.87 | ns | 6.83 | ns | 8.00 |
| **1,2,3,6,7,8-HxCDF** | ns | -3.44 | ns | 1.23 | ns | -3.79 | ns | -1.26 | ns | -0.96 |
| **2,3,4,6,7,8-HxCDF** | ns | -3.71 | ns | 4.32 | ns | -1.85 | ns | 0.90 | ns | 0.21 |
| **1,2,3,7,8,9-HxCDF** | ns | -9.60 | ns | -5.10 | ns | -5.40 | ns | -7.32 | ns | -6.89 |
| **1,2,3,4,6,7,8-HpCDF** | ns | -12.64 | ns | 1.69 | s | -7.14 | ns | -3.19 | ns | -2.76 |
| **1,2,3,4,7,8,9-HpCDF** | ns | -5.15 | ns | 0.22 | ns | -3.20 | ns | -4.25 | ns | 0.84 |
| **OCDF** | ns | 39.43 | ns | 46.54 | ns | 31.52 | ns | 20.71 | ns | 19.18 |
| **SUM** | ns | 7.76 | ns | 18.00 | ns | 8.22 | ns | 9.74 | ns | 52.16 |

### Table S8 - Natural attenuation effect: C-T0 vs C-T4 (significant reduction in grey, ns: not significant, s: significant)

| | PCDD/F concentration reduction (%): C-T0 vs C-T4 ($NA_{T4}$) | | | | | | | | | |
|---|---|---|---|---|---|---|---|---|---|---|
| | C1-4-T0 vs C2-T4 | | C1-4-T0 vs C3-T4 | | C1-4-T0 vs C4-T4 | | C5-T0 vs C5-T4 | | C7-T0 vs C7-T4 | |
| **2,3,7,8-TCDD** | ns | -14.14 | ns | -11.64 | ns | -9.89 | s | -29.30 | ns | -8.52 |
| **1,2,3,7,8-PCDD** | ns | -3.38 | ns | 2.25 | ns | -11.27 | ns | -3.87 | ns | 6.56 |
| **1,2,3,4,7,8-HxCDD** | ns | -2.64 | ns | -9.60 | ns | -10.48 | ns | -22.11 | ns | -3.44 |
| **1,2,3,6,7,8-HxCDD** | ns | -12.10 | ns | -7.94 | ns | -5.01 | ns | -20.24 | ns | -7.07 |
| **1,2,3,7,8,9-HxCDD** | ns | -12.87 | ns | -1.93 | ns | -9.75 | ns | -18.68 | s | -19.24 |
| **1,2,3,4,6,7,8-HpCDD** | s | -13.71 | ns | -2.47 | s | -6.86 | ns | -22.67 | s | -12.07 |
| **OCDD** | ns | -20.58 | ns | 10.69 | ns | -6.85 | ns | -42.50 | ns | -4.97 |
| **2,3,7,8-TCDF** | s | -10.96 | ns | 0.33 | ns | 13.70 | s | -16.09 | s | -10.97 |
| **1,2,3,7,8-PCDF** | ns | -8.27 | ns | 5.82 | ns | 10.59 | s | -16.86 | s | -7.02 |
| **2,3,4,7,8-PCDF** | ns | -10.43 | ns | 4.02 | ns | 29.08 | s | -15.79 | ns | -5.93 |
| **1,2,3,4,7,8-HxCDF** | ns | -1.52 | ns | -2.56 | ns | 3.80 | ns | -17.62 | ns | 3.87 |
| **1,2,3,6,7,8-HxCDF** | ns | -2.17 | ns | -1.70 | ns | 5.28 | s | -18.84 | ns | 2.29 |
| **2,3,4,6,7,8-HxCDF** | ns | -5.81 | ns | -1.86 | ns | 10.62 | ns | -16.10 | ns | -1.44 |
| **1,2,3,7,8,9-HxCDF** | ns | 5.55 | ns | -0.74 | ns | 6.67 | ns | -18.11 | ns | 16.01 |
| **1,2,3,4,6,7,8-HpCDF** | ns | -4.03 | s | -7.78 | ns | -1.24 | s | -16.50 | ns | -4.07 |
| **1,2,3,4,7,8,9-HpCDF** | ns | 17.20 | ns | 1.12 | ns | -6.18 | ns | -10.86 | ns | 23.24 |
| **OCDF** | ns | -3.54 | s | -13.18 | ns | -7.30 | ns | -20.40 | s | -14.46 |
| **SUM** | ns | -9.00 | ns | -0.25 | ns | 0.29 | ns | -25.96 | ns | -5.68 |

## RHIZOREMEDIATION EFFECT

### Table S9 - Rhizoremediation effect: C-T2 vs P-T2 (significant reduction in grey, ns: not significant, s: significant)

| | PCDD/F concentration reduction (%): C-T2 vs P-T2 ($RR_{T2}$) | | | | | | | | | | | | | | | | | | | |
|---|---|---|---|---|---|---|---|---|---|---|---|---|---|---|---|---|---|---|---|---|
| | C2-T2 vs P1-T2 | | C2-T2 vs P3-T2 | | C2-T2 vs P4-T2 | | C2-T2 vs P7-T2 | | C2-T2 vs P9-T2 | | C3-T2 vs P2-T2 | | C3-T2 vs P5-T2 | | C4-T2 vs P10-T2 | | C5-T2 vs P6-T2 | | C7-T2 vs P8-T2 | |
| **2,3,7,8-TCDD** | ns | 33.96 | ns | 16.04 | ns | 21.12 | ns | 15.51 | ns | 21.93 | ns | -17.42 | ns | -15.15 | ns | 0.23 | ns | -1.67 | ns | 5.99 |
| **1,2,3,7,8-PCDD** | ns | 1.39 | ns | 8.64 | ns | 4.42 | ns | 6.27 | ns | 5.57 | ns | -18.67 | ns | -26.08 | ns | 7.96 | ns | -28.55 | ns | -2.56 |
| **1,2,3,4,7,8-HxCDD** | ns | 7.78 | ns | 6.73 | ns | 7.00 | ns | -3.64 | ns | 9.87 | ns | -14.99 | ns | -25.33 | ns | 8.55 | ns | -11.51 | ns | -6.55 |
| **1,2,3,6,7,8-HxCDD** | ns | 7.82 | ns | 3.48 | ns | 8.25 | ns | -2.08 | ns | 11.73 | ns | -31.18 | ns | -37.50 | ns | 3.26 | ns | -15.32 | ns | -4.30 |
| **1,2,3,7,8,9-HxCDD** | ns | 1.36 | ns | 0.74 | ns | 7.26 | ns | -1.45 | ns | 6.27 | ns | -24.81 | ns | -41.89 | ns | 4.68 | ns | -7.96 | ns | -4.34 |
| **1,2,3,4,6,7,8-HpCDD** | ns | 5.07 | ns | 4.88 | ns | 3.09 | ns | 5.61 | ns | 10.28 | ns | -20.57 | ns | -29.51 | ns | 3.43 | ns | -10.70 | ns | -11.96 |
| **OCDD** | ns | -10.40 | ns | -12.75 | ns | -12.68 | ns | 36.81 | ns | 2.10 | ns | 22.99 | ns | -18.43 | ns | -13.83 | ns | -39.21 | ns | -60.59 |
| **2,3,7,8-TCDF** | ns | 10.01 | ns | -0.70 | ns | 1.20 | ns | 6.42 | ns | 21.06 | ns | 4.65 | ns | 1.41 | ns | 4.42 | ns | -22.47 | ns | -0.50 |
| **1,2,3,7,8-PCDF** | ns | 5.41 | ns | 1.38 | ns | 5.47 | ns | 22.20 | ns | 13.71 | ns | 5.93 | ns | -1.30 | ns | 3.66 | ns | -16.53 | ns | -0.90 |
| **2,3,4,7,8-PCDF** | ns | 3.45 | ns | 3.20 | ns | 4.71 | ns | 2.90 | ns | 22.00 | ns | 5.40 | ns | -1.45 | ns | 3.17 | ns | -16.01 | ns | -2.32 |
| **1,2,3,4,7,8-HxCDF** | ns | 7.34 | ns | 1.00 | ns | 1.73 | ns | 10.41 | ns | 12.78 | ns | 1.91 | ns | -3.74 | ns | 4.85 | ns | -18.07 | ns | -0.15 |
| **1,2,3,6,7,8-HxCDF** | ns | 8.38 | ns | 0.11 | ns | -2.16 | ns | 8.85 | ns | 8.21 | ns | 4.33 | ns | -2.08 | ns | 6.01 | ns | -14.54 | ns | 0.99 |
| **2,3,4,6,7,8-HxCDF** | ns | 5.46 | ns | 1.02 | ns | -1.27 | ns | 4.21 | ns | 11.66 | ns | 2.55 | ns | -5.40 | ns | 0.75 | ns | -16.98 | ns | -4.12 |
| **1,2,3,7,8,9-HxCDF** | ns | 4.34 | ns | -0.18 | ns | 0.56 | ns | 19.98 | ns | 13.23 | ns | 13.29 | ns | -0.27 | ns | 3.50 | ns | -11.77 | ns | 3.11 |
| **1,2,3,4,6,7,8-HpCDF** | ns | 6.86 | ns | 4.27 | ns | 4.45 | ns | 6.37 | ns | 13.87 | ns | -2.55 | ns | -7.81 | ns | 2.16 | ns | -16.50 | ns | -1.49 |
| **1,2,3,4,7,8,9-HpCDF** | ns | -0.13 | ns | 3.03 | ns | -2.11 | ns | 1.56 | ns | 17.65 | ns | 6.59 | ns | -2.39 | ns | 5.35 | ns | -13.43 | ns | 3.01 |
| **OCDF** | ns | -15.91 | ns | -17.96 | ns | -16.68 | ns | -13.22 | ns | -1.81 | ns | -9.63 | ns | -16.36 | ns | 5.14 | ns | 0.16 | ns | -3.11 |
| **SUM** | ns | -4.93 | ns | -8.29 | ns | -7.53 | ns | 10.17 | ns | 6.65 | ns | 4.73 | ns | -11.78 | ns | -0.65 | ns | -20.47 | ns | -29.85 |

### Table S10 - Rhizoremediation effect: C-T4 vs P-T4 (significant reduction in grey, ns: not significant, s: significant)

| | PCDD/F concentration reduction (%): C-T4 vs P-T4 ($RR_{T4}$) | | | | | | | | | | | | | | | | | | | |
|---|---|---|---|---|---|---|---|---|---|---|---|---|---|---|---|---|---|---|---|---|
| | C2-T4 vs P1-T4 | | C2-T4 vs P3-T4 | | C2-T4 vs P4-T4 | | C2-T4 vs P7-T4 | | C2-T4 vs P9-T4 | | C3-T4 vs P2-T4 | | C3-T4 vs P5-T4 | | C4-T4 vs P10-T4 | | C5-T4 vs P6-T4 | | C7-T4 vs P8-T4 | |
| **2,3,7,8-TCDD** | ns | 8.16 | ns | -11.37 | ns | -0.29 | ns | 18.08 | ns | 13.99 | ns | 11.05 | ns | -0.85 | ns | 13.33 | ns | 12.10 | ns | 18.01 |
| **1,2,3,7,8-PCDD** | ns | -5.01 | ns | -13.39 | ns | -1.12 | ns | 6.22 | ns | 1.73 | ns | -0.08 | ns | -3.88 | ns | 0.00 | ns | -13.68 | ns | 2.74 |
| **1,2,3,4,7,8-HxCDD** | ns | -8.05 | ns | -18.43 | ns | -18.75 | ns | -3.50 | ns | -0.11 | ns | -1.88 | ns | 1.03 | ns | 2.53 | ns | -13.13 | ns | 14.17 |
| **1,2,3,6,7,8-HxCDD** | ns | 10.60 | ns | -5.08 | ns | 12.31 | ns | 5.95 | ns | 8.23 | ns | 5.80 | ns | -7.20 | ns | -1.73 | ns | -5.50 | ns | 13.76 |
| **1,2,3,7,8,9-HxCDD** | ns | 5.63 | ns | 1.30 | ns | 16.57 | ns | 10.19 | ns | 18.93 | ns | 4.41 | ns | -2.17 | ns | 6.91 | ns | 1.59 | ns | 17.90 |
| **1,2,3,4,6,7,8-HpCDD** | ns | 5.25 | ns | -0.97 | ns | 9.34 | ns | 7.16 | ns | 5.76 | ns | -5.04 | ns | -8.92 | ns | 4.15 | ns | -5.25 | ns | 6.43 |
| **OCDD** | ns | 19.49 | ns | 2.77 | ns | 4.34 | ns | 2.31 | ns | 7.24 | ns | -19.11 | ns | -23.28 | ns | 20.70 | ns | 48.86 | ns | 1.74 |
| **2,3,7,8-TCDF** | ns | -4.37 | ns | -0.33 | ns | 2.26 | ns | 1.73 | ns | 4.54 | ns | -8.12 | ns | -7.04 | ns | -19.24 | ns | 9.03 | ns | 11.98 |
| **1,2,3,7,8-PCDF** | ns | -4.45 | ns | -11.32 | ns | -4.14 | ns | 2.67 | ns | 4.51 | ns | -11.98 | ns | -15.98 | ns | -14.40 | ns | 3.90 | ns | 8.28 |
| **2,3,4,7,8-PCDF** | ns | 0.31 | ns | -6.15 | ns | -2.42 | ns | 4.15 | ns | 9.62 | ns | -11.69 | ns | -14.84 | ns | -26.96 | ns | 2.57 | ns | 10.95 |
| **1,2,3,4,7,8-HxCDF** | ns | -9.19 | ns | -13.39 | ns | -9.25 | ns | -2.47 | ns | -3.27 | ns | -1.72 | ns | -6.04 | ns | -7.82 | ns | 0.16 | ns | -8.09 |
| **1,2,3,6,7,8-HxCDF** | ns | -9.42 | ns | -14.07 | ns | -7.44 | ns | -4.45 | ns | -3.50 | ns | -0.75 | ns | -6.38 | ns | -7.19 | ns | 0.74 | ns | -6.19 |
| **2,3,4,6,7,8-HxCDF** | ns | -4.02 | ns | -9.75 | ns | -4.52 | ns | 2.18 | ns | 3.08 | ns | -2.19 | ns | -5.80 | ns | -12.62 | ns | -1.30 | ns | -1.69 |
| **1,2,3,7,8,9-HxCDF** | ns | -14.08 | ns | -18.60 | ns | -14.19 | ns | -10.38 | ns | -3.83 | ns | -3.91 | ns | -8.39 | ns | -8.84 | ns | 0.46 | ns | -21.13 |
| **1,2,3,4,6,7,8-HpCDF** | ns | -3.48 | ns | -8.32 | ns | -2.74 | ns | 0.32 | ns | -1.21 | ns | 3.70 | ns | 0.18 | ns | -1.28 | ns | -3.22 | ns | -0.17 |
| **1,2,3,4,7,8,9-HpCDF** | ns | -19.77 | ns | -25.76 | ns | -20.68 | ns | -17.05 | ns | -15.46 | ns | -4.54 | ns | -8.72 | ns | 6.48 | ns | -9.68 | ns | -25.62 |
| **OCDF** | ns | -6.12 | s | -20.31 | ns | -2.55 | ns | 3.89 | ns | -2.84 | ns | 9.84 | ns | 5.02 | ns | 11.77 | ns | 6.16 | ns | 14.25 |
| **SUM** | ns | 0.02 | s | -9.27 | ns | -2.06 | ns | 0.96 | ns | 1.30 | ns | -6.59 | ns | -10.25 | ns | 1.69 | ns | 14.71 | ns | 3.27 |

# OVERALL EFFECT

## Table S11 - Overall effect: C-T0 vs P-T2 (significant reduction in grey, ns: not significant, s: significant)

| | PCDD/F concentration reduction (%): C-T0 vs P-T2 ($NA_{T2} + RR_{T2}$) | | | | | | | | | | | | | | | | | | | |
|---|---|---|---|---|---|---|---|---|---|---|---|---|---|---|---|---|---|---|---|---|
| | C1-4-T0 vs P1-T2 | | C1-4-T0 vs P3-T2 | | C1-4-T0 vs P4-T2 | | C1-4-T0 vs P7-T2 | | C1-4-T0 vs P9-T2 | | C1-4-T0 vs P2-T2 | | C1-4-T0 vs P5-T2 | | C1-4-T0 vs P10-T2 | | C5-T0 vs P6-T2 | | C7-T2 vs P8-T2 | |
| **2,3,7,8-TCDD** | ns | 25.41 | ns | 8.64 | ns | 13.39 | ns | 8.14 | ns | 14.14 | ns | 9.14 | ns | 12.14 | ns | 10.64 | ns | 32.20 | ns | 20.74 |
| **1,2,3,7,8-PCDD** | ns | 3.30 | ns | 10.68 | ns | 6.39 | ns | 8.26 | ns | 7.55 | ns | 13.61 | ns | 3.26 | ns | 0.71 | ns | -16.46 | ns | 14.53 |
| **1,2,3,4,7,8-HxCDD** | ns | 0.76 | ns | -0.22 | ns | 0.04 | ns | -9.91 | ns | 2.72 | ns | 16.13 | ns | 2.00 | ns | 0.81 | ns | -7.50 | ns | 5.57 |
| **1,2,3,6,7,8-HxCDD** | ns | 3.49 | ns | -0.68 | ns | 3.90 | ns | -6.02 | ns | 7.24 | ns | 15.21 | ns | 4.64 | ns | 3.38 | ns | -7.18 | ns | 1.37 |
| **1,2,3,7,8,9-HxCDD** | ns | 12.51 | ns | 11.82 | ns | 19.06 | ns | 9.39 | ns | 17.96 | ns | 35.28 | ns | 4.55 | ns | 14.32 | ns | 5.45 | ns | 8.62 |
| **1,2,3,4,6,7,8-HpCDD** | ns | -0.80 | ns | -0.97 | ns | -2.67 | ns | -0.29 | ns | 4.12 | ns | 12.68 | ns | -0.01 | ns | 2.61 | ns | -14.18 | ns | -4.68 |
| **OCDD** | ns | -5.40 | ns | -7.88 | ns | -7.81 | ns | 44.44 | ns | 7.80 | ns | 47.50 | ns | -2.18 | ns | -7.79 | ns | -31.57 | ns | 10.49 |
| **2,3,7,8-TCDF** | s | -11.59 | s | -20.20 | s | -18.67 | s | -14.47 | ns | -2.71 | s | -9.90 | s | -12.69 | ns | -9.93 | s | -28.70 | s | -11.20 |
| **1,2,3,7,8-PCDF** | ns | 7.19 | ns | 3.08 | ns | 7.25 | ns | 24.26 | ns | 15.62 | ns | 14.03 | ns | 6.25 | ns | 10.86 | ns | -4.92 | s | 8.13 |
| **2,3,4,7,8-PCDF** | ns | 13.96 | ns | 13.69 | ns | 15.35 | ns | 13.36 | ns | 34.40 | ns | 22.33 | ns | 14.38 | ns | 17.15 | ns | 4.17 | ns | 13.36 |
| **1,2,3,4,7,8-HxCDF** | ns | 7.23 | ns | 0.90 | ns | 1.63 | ns | 10.30 | ns | 12.66 | ns | 10.42 | ns | 4.29 | ns | 6.80 | ns | -12.48 | ns | 7.83 |
| **1,2,3,6,7,8-HxCDF** | ns | 4.65 | ns | -3.33 | ns | -5.53 | ns | 5.10 | ns | 4.48 | ns | 5.61 | ns | -0.88 | ns | 1.99 | ns | -15.61 | ns | 0.02 |
| **2,3,4,6,7,8-HxCDF** | ns | 1.56 | ns | -2.73 | ns | -4.93 | ns | 0.35 | ns | 7.52 | ns | 6.98 | ns | -1.31 | ns | -1.11 | ns | -16.24 | ns | -3.92 |
| **1,2,3,7,8,9-HxCDF** | ns | -5.68 | ns | -9.76 | ns | -9.09 | ns | 8.46 | ns | 2.36 | ns | 7.50 | ns | -5.36 | ns | -2.09 | s | -18.23 | ns | -4.00 |
| **1,2,3,4,6,7,8-HpCDF** | ns | -6.65 | s | -8.91 | ns | -8.76 | ns | -7.08 | ns | -0.53 | ns | -0.90 | ns | -6.26 | ns | -5.14 | s | -19.17 | ns | -4.21 |
| **1,2,3,4,7,8,9-HpCDF** | ns | -5.27 | ns | -2.27 | ns | -7.14 | ns | -3.67 | ns | 11.59 | ns | 6.82 | ns | -2.18 | ns | 1.98 | s | -17.10 | ns | 3.88 |
| **OCDF** | ns | 17.25 | ns | 14.39 | ns | 16.18 | ns | 21.01 | ns | 36.90 | ns | 32.43 | ns | 22.56 | ns | 38.27 | ns | 20.91 | ns | 15.47 |
| **SUM** | ns | 2.44 | ns | -1.17 | ns | -0.35 | ns | 18.72 | ns | 14.93 | ns | 23.59 | ns | 4.10 | ns | 7.52 | ns | -12.72 | ns | 6.74 |

## Table S12- Overall effect: C-T0 vs P-T4 (significant reduction in grey, ns: not significant, s: significant)

| | PCDD/F concentration reduction (%): C-T0 vs P-T4 ($NA_{T4} + RR_{T4}$) | | | | | | | | | | | | | | | | | | | |
|---|---|---|---|---|---|---|---|---|---|---|---|---|---|---|---|---|---|---|---|---|
| | C1-4-T0 vs P1-T4 | | C1-4-T0 vs P3-T4 | | C1-4-T0 vs P4-T4 | | C1-4-T0 vs P7-T4 | | C1-4-T0 vs P9-T4 | | C1-4-T0 vs P2-T4 | | C1-4-T0 vs P5-T4 | | C1-4-T0 vs P10-T4 | | C5-T0 vs P6-T4 | | C7-T2 vs P8-T4 | |
| **2,3,7,8-TCDD** | ns | -7.13 | s | -23.90 | ns | -14.39 | ns | 1.38 | ns | -2.13 | ns | -1.88 | ns | -12.39 | ns | 2.13 | ns | -20.74 | ns | 7.95 |
| **1,2,3,7,8-PCDD** | ns | -8.22 | ns | -16.32 | ns | -4.47 | ns | 2.63 | ns | -1.71 | ns | 2.17 | ns | -1.71 | ns | -11.27 | s | -17.02 | ns | 9.48 |
| **1,2,3,4,7,8-HxCDD** | ns | -10.48 | s | -20.59 | s | -20.90 | ns | -6.05 | ns | -2.75 | ns | -11.31 | ns | -8.68 | ns | -8.21 | s | -32.34 | ns | 10.25 |
| **1,2,3,6,7,8-HxCDD** | ns | -2.79 | s | -16.57 | ns | -1.28 | ns | -6.87 | ns | -4.87 | ns | -2.60 | s | -14.57 | ns | -6.65 | ns | -24.62 | ns | 5.72 |
| **1,2,3,7,8,9-HxCDD** | ns | -7.96 | ns | -11.74 | ns | 1.57 | ns | -3.99 | ns | 3.63 | ns | 2.39 | ns | -4.05 | ns | -3.51 | ns | -17.38 | ns | -4.78 |
| **1,2,3,4,6,7,8-HpCDD** | s | -9.18 | s | -14.55 | ns | -5.65 | s | -7.53 | ns | -8.74 | ns | -7.39 | ns | -11.17 | ns | -3.00 | ns | -26.73 | ns | -6.42 |
| **OCDD** | ns | -5.11 | ns | -18.39 | ns | -17.14 | ns | -18.75 | ns | -14.83 | ns | -10.46 | ns | -15.08 | ns | 12.44 | ns | -14.40 | ns | -3.32 |
| **2,3,7,8-TCDF** | s | -14.85 | s | -11.26 | ns | -8.95 | s | -9.42 | ns | -6.92 | s | -7.81 | ns | -6.73 | s | -8.18 | ns | -8.51 | ns | -0.30 |
| **1,2,3,7,8-PCDF** | s | -12.35 | s | -18.65 | s | -12.07 | ns | -5.82 | ns | -4.13 | ns | -6.85 | ns | -11.08 | ns | -5.33 | ns | -13.62 | ns | 0.68 |
| **2,3,4,7,8-PCDF** | s | -10.15 | s | -15.94 | s | -12.60 | ns | -6.72 | ns | -1.81 | ns | -8.14 | ns | -11.41 | ns | -5.72 | s | -13.62 | ns | 4.37 |
| **1,2,3,4,7,8-HxCDF** | s | -10.57 | s | -14.71 | s | -10.63 | ns | -3.95 | ns | -4.74 | ns | -4.24 | ns | -8.45 | ns | -4.32 | s | -17.49 | ns | -4.53 |
| **1,2,3,6,7,8-HxCDF** | s | -11.39 | s | -15.94 | s | -9.45 | s | -6.52 | ns | -5.60 | ns | -2.44 | ns | -7.97 | ns | -2.29 | s | -18.24 | ns | -4.04 |
| **2,3,4,6,7,8-HxCDF** | s | -9.59 | s | -14.99 | s | -10.06 | ns | -3.75 | ns | -2.91 | ns | -4.00 | ns | -7.54 | ns | -3.34 | s | -17.19 | ns | -3.10 |
| **1,2,3,7,8,9-HxCDF** | s | -9.31 | s | -14.08 | s | -9.43 | ns | -5.41 | ns | 1.50 | ns | -4.62 | ns | -9.07 | ns | -2.76 | s | -17.74 | ns | -8.51 |
| **1,2,3,4,6,7,8-HpCDF** | s | -7.37 | s | -12.02 | s | -6.66 | ns | -3.73 | ns | -5.19 | ns | -4.37 | ns | -7.61 | ns | -2.51 | s | -19.19 | ns | -4.23 |
| **1,2,3,4,7,8,9-HpCDF** | ns | -5.97 | s | -13.00 | ns | -7.04 | ns | -2.79 | ns | -0.92 | ns | -3.46 | ns | -7.70 | ns | -0.11 | s | -19.49 | ns | -8.34 |
| **OCDF** | ns | -9.45 | s | -23.13 | ns | -6.00 | ns | 0.21 | ns | -6.28 | ns | -4.64 | ns | -8.82 | ns | 3.61 | ns | -15.50 | ns | -2.27 |
| **SUM** | s | -8.97 | s | -17.44 | s | -10.88 | s | -8.12 | ns | -7.81 | ns | -6.82 | ns | -10.47 | ns | 1.99 | ns | -15.07 | ns | -2.60 |

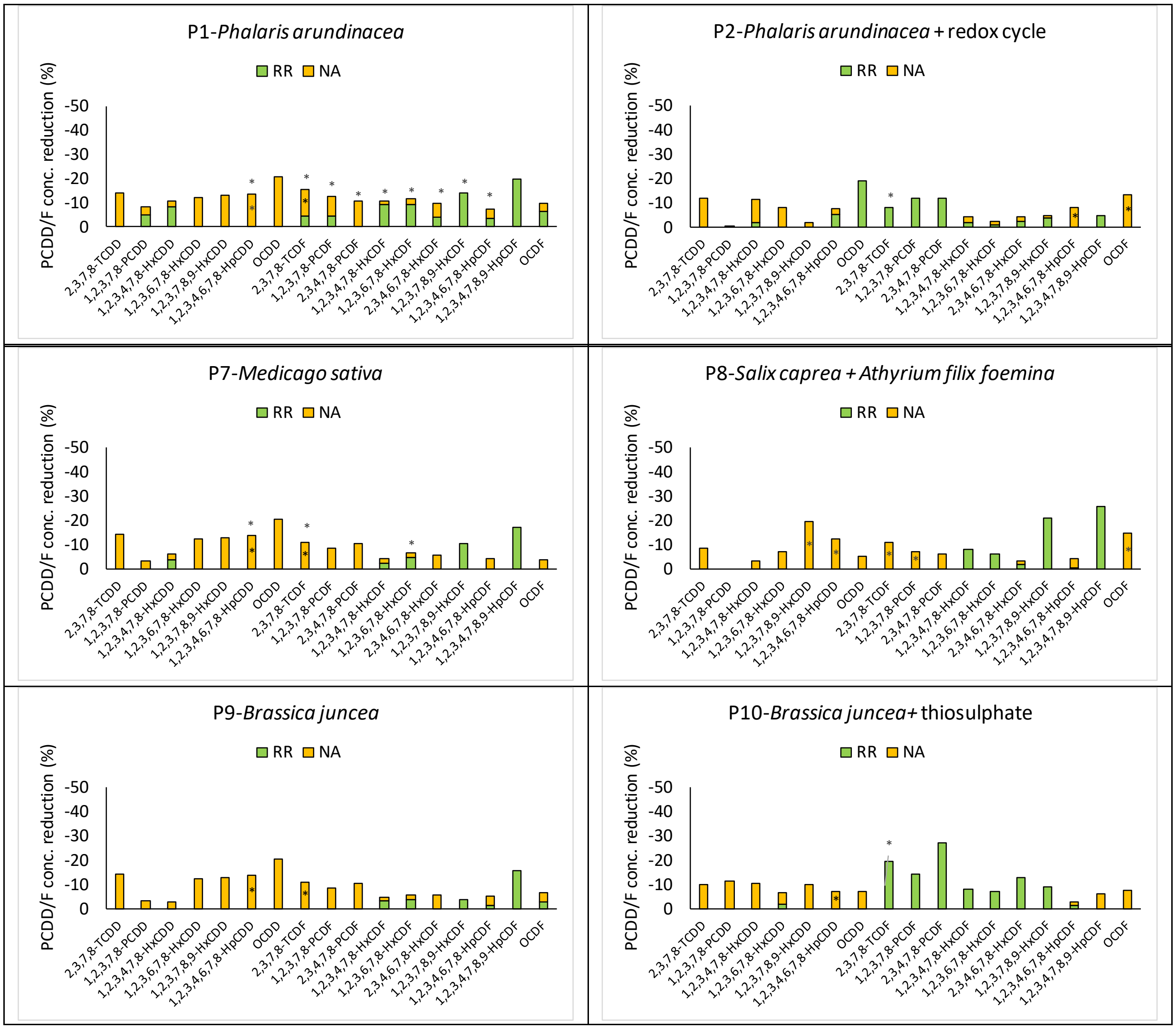


**Figure S4. Contribution of natural attenuation (NA) and rhizoremediation (RR) to the overall PCDD/F concentration reduction at T4.** Asterisks indicate statistically significant reduction for NA (when contained in the orange bar), for RR (when contained in the green bar), overall (when the asterisk is above the bar). (For interpretation of the references to color in this figure legend, the reader is referred to the web version of this article)

**CONTROL COMPARISON: UNFERTILIZED vs. FERTILIZED**

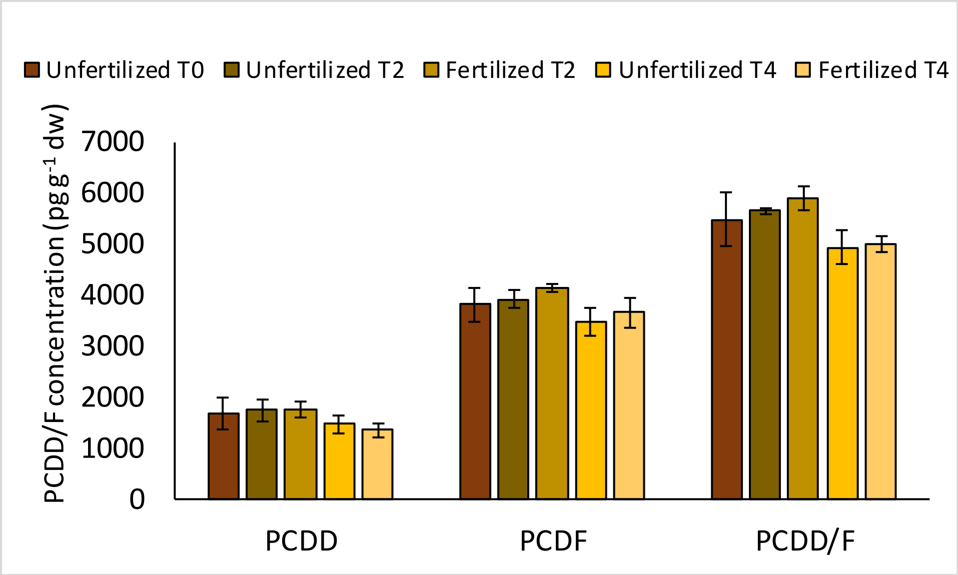


**Figure S5. PCDD/F concentrations in unfertilized and fertilized controls during the experiment (T0, T2 and T4)**

**HALF-LIVES**

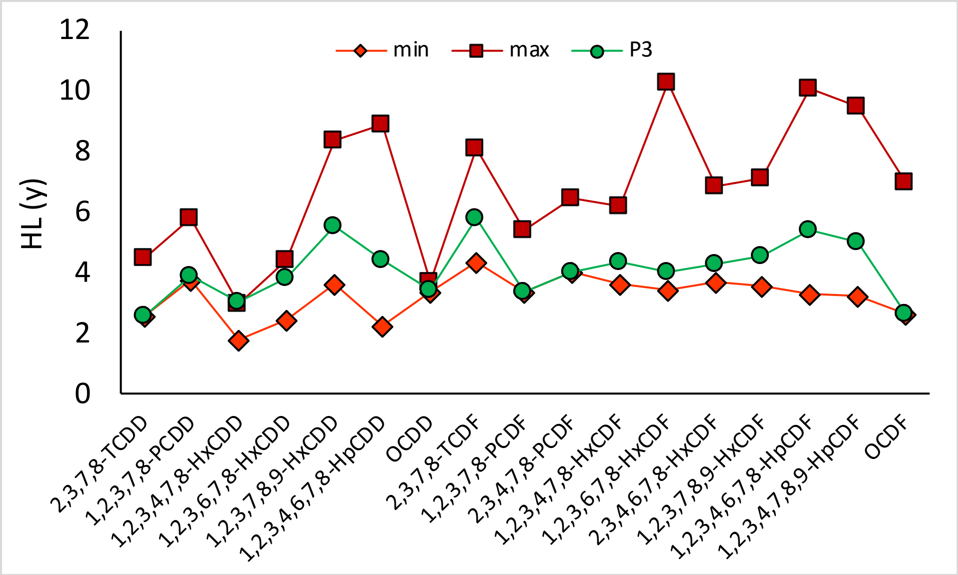


**Figure S6. PCDD/F half-lives in soil (years) at 25°C obtained from P3 and from other treatments (min and max)**

## Table S13- PCDD/F half-lives in soil at 25°C

| | PCDD/F half-lives in soil at 25°C | | | | | | | | | | | | | | | | | | | |
|---|---|---|---|---|---|---|---|---|---|---|---|---|---|---|---|---|---|---|---|---|
| | ONLY PLANT | | | | | | | | | | REDOX CYCLE | | | | COMPOST ADDITION | | ADDITIONAL SOIL | | THIOSULPHATE ADDITION | |
| | P1 | | P3 | | P4 | | P7 | | P9 | | P2 | | P5 | | P6 | | P8 | | P10 | |
| **PCDD/F congeners** | **HL** | ***p*** | **HL** | ***p*** | **HL** | ***p*** | **HL** | ***p*** | **HL** | ***p*** | **HL** | ***p*** | **HL** | ***p*** | **HL** | ***p*** | **HL** | ***p*** | **HL** | ***p*** |
| **2,3,7,8-TCDD** | 9.36 | 0.378 | 2.54 | 0.031 | 4.46 | 0.093 | nv | - | 32.21 | 0.780 | 36.55 | 0.803 | 5.24 | 0.131 | 2.98 | 0.109 | nv | - | nv | - |
| **1,2,3,7,8-PCDD** | 8.07 | 0.307 | 3.89 | 0.066 | 15.16 | 0.580 | nv | - | > 40 | 0.829 | nv | - | > 40 | 0.827 | 3.71 | 0.003 | nv | - | 5.79 | 0.164 |
| **1,2,3,4,7,8-HxCDD** | 6.26 | 0.131 | 3.00 | 0.004 | 2.95 | 0.018 | 11.11 | 0.333 | 24.88 | 0.668 | 5.77 | 0.111 | 7.63 | 0.175 | 1.77 | 0.003 | nv | - | 8.08 | 0.185 |
| **1,2,3,6,7,8-HxCDD** | 24.51 | 0.681 | 3.82 | 0.004 | > 40 | 0.842 | 9.73 | 0.316 | 13.88 | 0.441 | 26.34 | 0.679 | 4.40 | 0.009 | 2.45 | 0.098 | nv | - | 10.07 | 0.293 |
| **1,2,3,7,8,9-HxCDD** | 8.35 | 0.242 | 5.55 | 0.108 | nv | - | 17.03 | 0.562 | nv | - | nv | - | 16.73 | 0.588 | 3.63 | 0.225 | 14.13 | 0.619 | 19.41 | 0.612 |
| **1,2,3,4,6,7,8-HpCDD** | 7.20 | 0.004 | 4.41 | 0.004 | 11.91 | 0.271 | 8.85 | 0.009 | 7.57 | 0.122 | 9.02 | 0.162 | 5.85 | 0.057 | 2.23 | 0.195 | 10.44 | 0.069 | 22.76 | 0.556 |
| **OCDD** | 13.22 | 0.701 | 3.41 | 0.101 | 3.68 | 0.194 | 3.34 | 0.101 | 4.31 | 0.262 | 6.27 | 0.437 | 4.24 | 0.241 | 4.45 | 0.771 | 20.55 | 0.825 | nv | - |
| **2,3,7,8-TCDF** | 4.31 | 0.004 | 5.80 | 0.031 | 7.39 | 0.222 | 7.00 | 0.004 | 9.66 | 0.339 | 8.52 | 0.031 | 9.94 | 0.368 | 7.79 | 0.468 | > 40 | 0.938 | 8.12 | 0.004 |
| **1,2,3,7,8-PCDF** | 5.26 | 0.004 | 3.36 | 0.004 | 5.39 | 0.004 | 11.56 | 0.322 | 16.42 | 0.475 | 9.76 | 0.248 | 5.90 | 0.087 | 4.73 | 0.069 | nv | - | 12.64 | 0.364 |
| **2,3,4,7,8-PCDF** | 6.47 | 0.004 | 3.99 | 0.004 | 5.14 | 0.004 | 9.97 | 0.422 | 37.83 | 0.826 | 8.15 | 0.339 | 5.72 | 0.196 | 4.73 | 0.005 | nv | - | 11.76 | 0.497 |
| **1,2,3,4,7,8-HxCDF** | 6.20 | 0.004 | 4.35 | 0.004 | 6.17 | 0.004 | 17.18 | 0.464 | 14.25 | 0.401 | 15.99 | 0.425 | 7.85 | 0.160 | 3.60 | 0.029 | 14.93 | 0.314 | 15.70 | 0.415 |
| **1,2,3,6,7,8-HxCDF** | 5.73 | 0.004 | 3.99 | 0.004 | 6.98 | 0.004 | 10.27 | 0.009 | 12.03 | 0.302 | 28.01 | 0.615 | 8.34 | 0.166 | 3.44 | 0.028 | 16.80 | 0.376 | 29.86 | 0.639 |
| **2,3,4,6,7,8-HxCDF** | 6.87 | 0.004 | 4.27 | 0.004 | 6.53 | 0.004 | 18.12 | 0.511 | 23.49 | 0.637 | 16.96 | 0.484 | 8.83 | 0.236 | 3.67 | 0.011 | 21.99 | 0.476 | 20.38 | 0.559 |
| **1,2,3,7,8,9-HxCDF** | 7.09 | 0.009 | 4.56 | 0.004 | 6.99 | 0.009 | 12.45 | 0.288 | nv | - | 14.64 | 0.365 | 7.29 | 0.130 | 3.55 | 0.023 | 7.79 | 0.183 | 24.74 | 0.563 |
| **1,2,3,4,6,7,8-HpCDF** | 9.05 | 0.004 | 5.41 | 0.004 | 10.04 | 0.009 | 18.23 | 0.496 | 12.99 | 0.358 | 15.51 | 0.425 | 8.76 | 0.221 | 3.25 | 0.011 | 16.01 | 0.284 | 27.30 | 0.660 |
| **1,2,3,4,7,8,9-HpCDF** | 11.25 | 0.322 | 4.98 | 0.009 | 9.49 | 0.070 | 24.52 | 0.643 | > 40 | 0.898 | 19.65 | 0.602 | 8.65 | 0.254 | 3.20 | 0.019 | 7.95 | 0.311 | > 40 | 0.986 |
| **OCDF** | 6.98 | 0.062 | 2.63 | 0.004 | 11.20 | 0.184 | nv | - | 10.69 | 0.200 | 14.58 | 0.267 | 7.50 | 0.126 | 4.11 | 0.094 | 30.14 | 0.641 | nv | - |

**NOTE:** "nv" means that concentration at T4 was equal or slightly higher of the initial concentration showing no degradation in these conditions; "p" is the p-value

**BIODEGRADATION vs. BOUND RESIDUE FORMATION**

**Table S14 – Comparison (ratio) between PCDD/F concentrations in soil pore water ($C_{pw(1)}$) estimated from soil/water partition coefficient (Kd) and those ($C_{pw(2)}$) estimated from the root concentration factor**.

| PCDD/Fs | Cs (ng/kg dw) | Cr (ng/kg dw) | Log Kow | Kd (L/kg) | $C_{pw(1)}$ (ng/L) (**) | $C_{pw(2)}$ (ng/L) (***) | Ratio |
|---|---|---|---|---|---|---|---|
| 2,3,7,8-TCDD | 1.01 | <LOQ | 6.8 | 4.66E+04 | 2.18E-05 | n.a. | n.a. |
| 1,2,3,7,8-PCDD | 6.68 | <LOQ | 7.4 (*) | 1.85E+05 | 3.61E-05 | n.a. | n.a. |
| 1,2,3,4,7,8-HxCDD | 5.13 | <LOQ | 7.8 (*) | 4.66E+05 | 1.10E-05 | n.a. | n.a. |
| 1,2,3,6,7,8-HxCDD | 10.15 | 10.61 | 7.8 (*) | 4.66E+05 | 2.18E-05 | 6.58E-05 | 3.02 |
| 1,2,3,7,8,9-HxCDD | 8.58 | <LOQ | 7.8 (*) | 4.66E+05 | 1.84E-05 | n.a. | n.a. |
| 1,2,3,4,6,7,8-HpCDD | 77.71 | 52.47 | 8 | 7.38E+05 | 1.05E-04 | 2.28E-04 | 2.17 |
| OCDD | 1258.03 | 198.10 | 8.2 | 1.17E+06 | 1.08E-03 | 6.05E-04 | 0.56 |
| 2,3,7,8-TCDF | 558.70 | 607.18 | 6.1 | 9.29E+03 | 6.01E-02 | 7.67E-02 | 1.28 |
| 1,2,3,7,8-PCDF | 278.63 | 209.80 | 6.5 (*) | 2.33E+04 | 1.19E-02 | 1.30E-02 | 1.09 |
| 2,3,4,7,8-PCDF | 205.71 | 246.52 | 6.5 | 2.33E+04 | 8.81E-03 | 1.53E-02 | 1.74 |
| 1,2,3,4,7,8-HxCDF | 462.45 | 379.81 | 7 | 7.38E+04 | 6.27E-03 | 9.73E-03 | 1.55 |
| 1,2,3,6,7,8-HxCDF | 93.93 | 79.29 | 7 (*) | 7.38E+04 | 1.27E-03 | 2.03E-03 | 1.60 |
| 2,3,4,6,7,8-HxCDF | 63.12 | 58.96 | 7 (*) | 7.38E+04 | 8.55E-04 | 1.51E-03 | 1.77 |
| 1,2,3,7,8,9-HxCDF | 119.58 | 79.43 | 7 (*) | 7.38E+04 | 1.62E-03 | 2.04E-03 | 1.26 |
| 1,2,3,4,6,7,8-HpCDF | 252.07 | 147.82 | 7.4 | 1.85E+05 | 1.36E-03 | 1.86E-03 | 1.37 |
| 1,2,3,4,7,8,9-HpCDF | 139.00 | 60.78 | 7.4 (*) | 1.85E+05 | 7.50E-04 | 7.66E-04 | 1.02 |
| OCDF | 977.60 | 387.34 | 8 | 7.38E+05 | 1.32E-03 | 1.69E-03 | 1.27 |

**NOTE:** Cs is the concentration in soil, Cr is the concentration in roots, Log Kow is the octanol-water partition coefficient from [1](*) due to a lack of data the values refer to congeners with the same number of chlorines; (**) calculated from Kd and soil concentrations; (***) calculated from root concentrations and root concentration factor (RCF) obtained from [5]; n.a. means not available.

**Table S15 - Natural attenuation half-life for PCDD/F in soil from laboratory and field experiments with spiked and aged soil**

| Natural attenuation HL in soil (years) | | | | | | | | | | | |
|---|---|---|---|---|---|---|---|---|---|---|---|
| **References** | **A** | **B** | **C** | **D** | **E** | **F** | **G** | **H** | **I** | **J** | **K** |
| **Experiment type** | **Microcosm** | **Soil column** | **Microcosm** | **Microcosm** | **Microcosm** | **Selected values** | **Field** | **Field** | **Field** | **Field** | **Field** |
| **Contamination type** | **Spiked** | **Spiked** | **Spiked** | **Spiked** | **Aged** | | **Aged** | **Aged** | **Aged** | **Aged** | **Aged** |
| **Experiment time** | **1 y** | **1 y** | **1.2 y** | **0.3 y** | **0.2 y** | | **2 y** | **20 y** | **20 y** | **43 y** | **40 y** |
| **Temperature** | **28-30°C** | **20-35 °C** | **30°C** | **20°C** | **28°C** | **25°C** | **nr** | **nr** | **25°C** | **nr** | **nr** |
| **2,3,7,8-TCDD** | 1.4 | >1 | | | | 1-3 | >2 | 23 | 30 | | 5 |
| **1,2,3,7,8-PCDD** | | | | | | 1-3 | >2 | 21 | 33 | | 17 |
| **1,2,3,4,7,8-HxCDD** | | | 2.9 | | | 3-11 | >2 | 22 | 79 | | 14 |
| **1,2,3,6,7,8-HxCDD** | | | | | | 3-11 | >2 | 21 | 18 | | 15 |
| **1,2,3,7,8,9-HxCDD** | | | | | | 3-11 | >2 | 22 | 23 | | 11 |
| **1,2,3,4,6,7,8-HpCDD** | | | 2.9 | | | 3-11 | >2 | 21 | 30 | | 13 |
| **OCDD** | | | | 1.7 | | 3-11 | >2 | 20 | 43 | 14 | 12 |
| **2,3,7,8-TCDF** | | | | | | 1-3 | >2 | 27 | 18 | | 25 |
| **1,2,3,7,8-PCDF** | | | | | | 1-3 | >2 | 36 | 15 | | 12 |
| **2,3,4,7,8-PCDF** | | | | | | 1-3 | >2 | 24 | 18 | | 15 |
| **1,2,3,4,7,8-HxCDF** | | | | | | 1-3 | >2 | 29 | 20 | | 21 |
| **1,2,3,6,7,8-HxCDF** | | | | | | 1-3 | >2 | 38 | 23 | | 11 |
| **2,3,4,6,7,8-HxCDF** | | | | | | 1-3 | >2 | 41 | 15 | | 21 |
| **1,2,3,7,8,9-HxCDF** | | | | | | 1-3 | >2 | 18 | 16 | | |
| **1,2,3,4,6,7,8-HpCDF** | | | 2.5 | | 0.03 | 1-3 | >2 | | 11 | | 10 |
| **1,2,3,4,7,8,9-HpCDF** | | | | | 0.07 | 1-3 | >2 | 19 | 10 | | 7 |
| **OCDF** | | | | | 0.02 | 3-11 | >2 | 22 | 8 | | 11 |

A. Ref. [6] (calculated from concentrations and first order kinetics)
B. Ref. [7]
C. Ref. [8]
D. Ref. [9] (calculated from concentrations and first order kinetics)
E. Ref. [10] (calculated from concentration reduction and first order kinetics)
F. Ref. [1]
G. Ref. [11]
H. Ref. [12] (calculated from concentrations and first order kinetics)
I. Ref.[13] (converted to 25°C, sediments)
J. Ref. [14] (model)
K. Ref. [15]

**Table S16 - Bioaugmentation half-life for PCDD/F in soil from laboratory experiments (continues in the next page)**

| HL from bioaugmentation experiments (years) | | | | | |
|---|---|---|---|---|---|
| **Reference** | **A** | **B** | **C** | **D** | **E** |
| **Experiment type** | **Microcosm** | **Microcosm** | **Microcosm** | **Microcosm** | **Microcosm** |
| **Matrices** | **Medium** | **Medium with contaminated soil** | **Slurry with contaminated soil** | **Slurry with contaminated soil** | **Contaminated soil** |
| **Microorganism** | **White Rot Fungus** | **Bacteria** | **White Rot Fungus** | **Bacteria** | **White Rot Fungus** |
| **Species** | ***Phanerochaete sordida* YK-624** | ***Pseudomonas sp.* strain CA10** | ***Stropharia rugosoannulata*** | ***Pseudomonas mendocina* NSYSU** | ***Stropharia rugosoannulata*** |
| **Contamination type** | **Spiked** | **Aged** | **Aged** | **Aged** | **Aged** |
| **Experiment time** | **14 days** | **7 days** | **105 days** | **65 days** | **90 days** |
| **Temperature** | **30°C** | **30°C** | **nr** | **20°C** | **room temperature** |
| **2,3,7,8-TCDD** | 0.09 | 0.07 | | | |
| **1,2,3,7,8-PCDD** | 0.09 | 0.16 | 0.26 | | |
| **1,2,3,4,7,8-HxCDD** | 0.05 | 0.18 | 0.48 | | |
| **1,2,3,6,7,8-HxCDD** | | 0.12 | 0.26 | | 0.23 |
| **1,2,3,7,8,9-HxCDD** | | 0.17 | 0.33 | | 0.27 |
| **1,2,3,4,6,7,8-HpCDD** | 0.06 | 0.14 | 0.29 | | 0.28 |
| **OCDD** | 0.17 | 0.30 | 0.28 | 0.11 | 0.37 |
| **2,3,7,8-TCDF** | 0.05 | 0.11 | 0.09 | | 3.33 |
| **1,2,3,7,8-PCDF** | 0.10 | 0.11 | 0.25 | | 0.38 |
| **2,3,4,7,8-PCDF** | | 0.13 | | | 0.17 |
| **1,2,3,4,7,8-HxCDF** | 0.04 | 0.33 | 0.42 | | 0.18 |
| **1,2,3,6,7,8-HxCDF** | | 0.13 | 0.58 | | 0.12 |
| **2,3,4,6,7,8-HxCDF** | | 0.41 | 0.42 | | 0.18 |
| **1,2,3,7,8,9-HxCDF** | | | 0.15 | | |
| **1,2,3,4,6,7,8-HpCDF** | 0.05 | | 1.32 | | 0.16 |
| **1,2,3,4,7,8,9-HpCDF** | | 0.18 | 0.22 | | 0.27 |
| **OCDF** | 0.06 | 3.32 | 0.69 | | 0.16 |
| **SUM** | | | | | |

A. Ref. [16] (calculated from concentration reduction and first order kinetics)
B. Ref. [17] (calculated from concentration reduction and first order kinetics)
C. Ref. [18] (calculated from concentration reduction and first order kinetics)
D. Ref. [19] (calculated from concentration reduction and first order kinetics)
E. Ref. [20] (calculated from concentration reduction and first order kinetics)

## Table S16 - Bioaugmentation half-life for PCDD/F in soil from laboratory experiments (continued)

| HL from bioaugmentation experiments (years) | | | | | |
|---|---|---|---|---|---|
| **Reference** | **F** | **G** | **H** | **I** | **J** |
| **Experiment type** | **Microcosm** | **Microcosm** | **Microcosm** | **Composting reactor** | **Hypoxic reactor** |
| **Matrices** | **Contaminated soil** | **Medium with contaminated soil** | **Medium** | **Contaminated soil** | **Contaminated soil** |
| **Microorganism** | **From compost (waste sludge)** | **Bacteria** | **White Rot Fungus** | **From compost (food waste)** | **From compost (cow dung)** |
| **Species** | **Different species** | ***Pseudomonas mendocina* NSYSU** | ***Rigidoporus* sp.** | **Different species** | **Different species** |
| **Contamination type** | **Aged** | **Aged** | **Spiked** | **Aged** | **Aged** |
| **Experiment time** | **30 days** | **64 days** | **28 days** | **42 days** | **14-128 days** |
| **Temperature** | **room temperature** | **20 °C (anaerobic)** | **nr** | **30 - > 70°C** | 25-28°C |
| **2,3,7,8-TCDD** | | | 0.05 | | |
| **1,2,3,7,8-PCDD** | | | | | |
| **1,2,3,4,7,8-HxCDD** | | | | | |
| **1,2,3,6,7,8-HxCDD** | 0.04 | | | | |
| **1,2,3,7,8,9-HxCDD** | 0.16 | | | | |
| **1,2,3,4,6,7,8-HpCDD** | 0.06 | | | | |
| **OCDD** | 0.07 | | | | 0.08 - 0.47 |
| **2,3,7,8-TCDF** | | | | | |
| **1,2,3,7,8-PCDF** | | | | | |
| **2,3,4,7,8-PCDF** | | | | | |
| **1,2,3,4,7,8-HxCDF** | 0.10 | | | | |
| **1,2,3,6,7,8-HxCDF** | 0.54 | | | | |
| **2,3,4,6,7,8-HxCDF** | 0.10 | | | | |
| **1,2,3,7,8,9-HxCDF** | | | | | |
| **1,2,3,4,6,7,8-HpCDF** | | | | | |
| **1,2,3,4,7,8,9-HpCDF** | 0.08 | | | | |
| **OCDF** | 0.05 | 0.14 | | | 0.06-0.31 |
| **SUM** | | | | 0.07 | |

F. Ref. [21] (calculated from concentration reduction and first order kinetics)
G. Ref. [22] (calculated from concentration reduction and first order kinetics)
H. Ref. [23] (calculated from concentration reduction and first order kinetics)
I. Ref. [24] (calculated from degradation rate)
J. Ref. [25] (calculated from degradation rate)

## Table S17 - Rhizoremediation half-life for selected PCDD/F in soil

| **HL from rhizoremediation experiment (years)** | | |
|---|---|---|
| **Reference** | **A** | **B** |
| **Experiment type** | Pot | Pot |
| **Plant species** | *Cynodon dactylon, Agrostis palustris* Huds.*, Zoysia japonica, Trifolium repens* L. | *Trifolium repens* L. |
| **Contamination type** | Spiked soil | Spiked soil |
| **Experiment time** | 60 days | 84 days |
| **Temperature** | 26°C | 26°C |
| DD (dibenzo-p-dioxin) | | 0.61 |
| 1-CDD (1-chlorodibenzo-p-dioxin) | | 0.71 |
| 2,7-DCDD (2,7-dichlorodibenzo-p-dioxin) | | ns |
| 1,2,4-TCDD (1,2,4-trichlorodibenzo-p-dioxin) | | ns |
| 1,2,3,4-TCDD (1,2,3,4-tetrachlorodibenzo-p-dioxin) | | ns |
| DF (dibenzofuran) | 0.1-0.3 | 0.33 |
| 2,8-DCDF (2,8-Dichlorodibenzofuran) | | ns |
| 2,4,8-TCDF (2,4,8-Trichlorodibenzofuran) | | ns |

A. Ref. [26] (calculated from concentration reduction and first order kinetics)
B. Ref. [27] (calculated from concentration reduction and first order kinetics)
ns. Concentration reduction not statistically significant with respect to control

**REFERENCES**


(1) Mackay, D.; Shiu, W. Y.; Ma, Kuo-Ching; Lee, S. C. *Handbook of Physical-Chemical Properties and Environmental Fate for Organic Chemicals*, 2nd ed.; CRC/Taylor & Francis: Boca Raton, FL, 2006.

(2) Bolinder, M. A.; Angers, D. A.; Bélanger, G.; Michaud, R.; Laverdière, M. R. Root Biomass and Shoot to Root Ratios of Perennial Forage Crops in Eastern Canada. *Canadian Journal of Plant Science* **2002**, *82* (4), 731–737. https://doi.org/10.4141/P01-139.

(3) Woodward, F. I. The Significance of Interspecific Differences in Specific Leaf Area to the Growth of Selected Herbaceous Species from Different Altitudes. *New Phytologist* **1983**, *95* (2), 313–323.

(4) Ghirardello, D.; Morselli, M.; Semplice, M.; Di Guardo, A. A Dynamic Model of the Fate of Organic Chemicals in a Multilayered Air/Soil System: Development and Illustrative Application. *Environmental science & technology* **2010**, *44* (23), 9010–9017.

(5) Briggs, G. G.; Bromilow, R. H.; Evans, A. A. Relationships between Lipophilicity and Root Uptake and Translocation of Non-Ionised Chemicals by Barley. *Pestic. Sci.* **1982**, *13* (5), 495–504. https://doi.org/10.1002/ps.2780130506.

(6) Kearney, P. C.; Woolson, E. A.; Ellington, C. P. Persistence and Metabolism of Chlorodioxins in Soils. *Environ. Sci. Technol.* **1972**, *6* (12), 1017–1019. https://doi.org/10.1021/es60071a010.

(7) Kapila, S.; Yanders, A. F.; Orazio, C. E.; Meadows, J. E.; Cerlesi, S.; Clevenger, T. E. Field and Laboratory Studies on the Movement and Fate of Tetrachlorodibenzo-p-Dioxin in Soil. *Chemosphere* **1989**, *18* (1–6), 1297–1304. https://doi.org/10.1016/0045-6535(89)90268-3.

(8) Adriaens, P.; Grbic'-Galic, D. Reductive Dechlorination of PCDD/F by Anaerobic Cultures and Sediments. *Chemosphere* **1994**, *29* (9–11), 2253–2259. https://doi.org/10.1016/0045-6535(94)90392-1.

(9) Brodsky, J.; Brodesser, J.; Bauer, C.; Römbke, J. The Environmental Fate of Six Existing Chemicals in Laboratory Tests. *Chemosphere* **1997**, *34* (3), 515–538. https://doi.org/10.1016/S0045-6535(96)00390-6.

(10) Chen, W.-Y.; Wu, J.-H.; Lin, Y.-Y.; Huang, H.-J.; Chang, J.-E. Bioremediation Potential of Soil Contaminated with Highly Substituted Polychlorinated Dibenzo-p-Dioxins and Dibenzofurans: Microcosm Study and Microbial Community Analysis. *Journal of Hazardous Materials* **2013**, *261*, 351–361. https://doi.org/10.1016/j.jhazmat.2013.07.039.

(11) Hagenmaier, H.; She, J.; Lindig, C. Persistence of Polychlorinated Dibenzo-p-Dioxins and Polychlorinated Dibenzofurans in Contaminated Soil at Maulach and Rastatt in Southwest Germany. *Chemosphere* **1992**, *25* (7–10), 1449–1456. https://doi.org/10.1016/0045-6535(92)90168-Q.

(12) McLachlan, M. S.; Sewart, A. P.; Bacon, J. R.; Jones, K. C. Persistence of PCDD/Fs in a Sludge-Amended Soil. *Environ. Sci. Technol.* **1996**, *30* (8), 2567–2571. https://doi.org/10.1021/es950932g.

(13) Sinkkonen, S.; Paasivirta, J. Degradation Half-Life Times of PCDDs, PCDFs and PCBs for Environmental Fate Modeling. *Chemosphere* **2000**, *40* (9–11), 943–949. https://doi.org/10.1016/S0045-6535(99)00337-9.

(14) Zhao, X.; Zheng, M.; Zhang, B.; Qian, Y.; Xu, X. Estimation of OCDD Degradation Rate in Soil. *J Environ Sci (China)* **2005**, *17* (6), 981–983.

(15) Seike, N.; Kashiwagi, N.; Otani, T. PCDD/F Contamination over Time in Japanese Paddy Soils. *Environ. Sci. Technol.* **2007**, *41* (7), 2210–2215. https://doi.org/10.1021/es062318i.

(16) Takada, S.; Nakamura, M.; Matsueda, T.; Kondo, R.; Sakai, K. Degradation of Polychlorinated Dibenzo-p-Dioxins and Polychlorinated Dibenzofurans by the White Rot Fungus Phanerochaete Sordida YK-624. *APPL. ENVIRON. MICROBIOL.* **1996**, *62*, 6.

(17) Habe, H.; Chung, J.-S.; Lee, J.-H.; Kasuga, K.; Yoshida, T.; Nojiri, H.; Omori, T. Degradation of Chlorinated Dibenzofurans and Dibenzo-p-Dioxins by Two Types of Bacteria Having Angular

Dioxygenases with Different Features. *Applied and Environmental Microbiology* **2001**, *67* (8), 3610–3617. https://doi.org/10.1128/AEM.67.8.3610-3617.2001.
(18) Valentín, L.; Oesch-Kuisma, H.; Steffen, K. T.; Kähkönen, M. A.; Hatakka, A.; Tuomela, M. Mycoremediation of Wood and Soil from an Old Sawmill Area Contaminated for Decades. *Journal of Hazardous Materials* **2013**, *260*, 668–675. https://doi.org/10.1016/j.jhazmat.2013.06.014.
(19) Tu, Y. T.; Liu, J. K.; Lin, W. C.; Lin, J. L.; Kao, C. M. Enhanced Anaerobic Biodegradation of OCDD-Contaminated Soils by Pseudomonas Mendocina NSYSU: Microcosm, Pilot-Scale, and Gene Studies. *Journal of Hazardous Materials* **2014**, *278*, 433–443. https://doi.org/10.1016/j.jhazmat.2014.06.014.
(20) Anasonye, F.; Winquist, E.; Kluczek-Turpeinen, B.; Räsänen, M.; Salonen, K.; Steffen, K. T.; Tuomela, M. Fungal Enzyme Production and Biodegradation of Polychlorinated Dibenzo-p-Dioxins and Dibenzofurans in Contaminated Sawmill Soil. *Chemosphere* **2014**, *110*, 85–90. https://doi.org/10.1016/j.chemosphere.2014.03.079.
(21) Chen, W.-Y.; Wu, J.-H.; Lin, S.-C.; Chang, J.-E. Bioremediation of Polychlorinated-p-Dioxins/Dibenzofurans Contaminated Soil Using Simulated Compost-Amended Landfill Reactors under Hypoxic Conditions. *Journal of Hazardous Materials* **2016**, *312*, 159–168. https://doi.org/10.1016/j.jhazmat.2016.03.060.
(22) Lin, J. BIOREMEDIATION OF OCDF-CONTAMINATED SOILS BY NOVEL BACTERIAL STRAIN. *Appl Ecol Env Res* **2017**, *15* (3), 713–723. https://doi.org/10.15666/aeer/1503_713723.
(23) Dao, A. T. N.; Vonck, J.; Janssens, T. K. S.; Dang, H. T. C.; Brouwer, A.; de Boer, T. E. Screening White-Rot Fungi for Bioremediation Potential of 2,3,7,8-Tetrachlorodibenzo-p-Dioxin. *Industrial Crops and Products* **2019**, *128*, 153–161. https://doi.org/10.1016/j.indcrop.2018.10.059.
(24) Huang, W.-Y.; Ngo, H.-H.; Lin, C.; Vu, C.-T.; Kaewlaoyoong, A.; Boonsong, T.; Tran, H.-T.; Bui, X.-T.; Vo, T.-D.-H.; Chen, J.-R. Aerobic Co-Composting Degradation of Highly PCDD/F-Contaminated Field Soil. A Study of Bacterial Community. *Science of The Total Environment* **2019**, *660*, 595–602. https://doi.org/10.1016/j.scitotenv.2018.12.312.
(25) Wu, J.-H.; Chen, W.-Y.; Kuo, H.-C.; Li, Y.-M. Redox Fluctuations Shape the Soil Microbiome in the Hypoxic Bioremediation of Octachlorinated Dibenzodioxin- and Dibenzofuran-Contaminated Soil. *Environmental Pollution* **2019**, *248*, 506–515. https://doi.org/10.1016/j.envpol.2019.02.053.
(26) Wang, Y.; Oyaizu, H. Evaluation of the Phytoremediation Potential of Four Plant Species for Dibenzofuran-Contaminated Soil. *Journal of Hazardous Materials* **2009**, *168* (2–3), 760–764. https://doi.org/10.1016/j.jhazmat.2009.02.082.
(27) Wang, Y.; Oyaizu, H. Enhanced Remediation of Dioxins-Spiked Soil by a Plant-Microbe System Using a Dibenzofuran-Degrading Comamonas Sp and Trifolium Repens L. *Chemosphere* **2011**, *85* (7), 1109–1114. https://doi.org/10.1016/j.chemosphere.2011.07.028.
